# New mutations in the core *Schizosaccharomyces pombe* spindle pole body scaffold Ppc89 reveal separable functions in regulating cell division

**DOI:** 10.1093/g3journal/jkae249

**Published:** 2024-10-29

**Authors:** Sarah M Hanna, Bita Tavafoghi, Jun-Song Chen, Isaac Howard, Liping Ren, Alaina H Willet, Kathleen L Gould

**Affiliations:** Department of Cell and Developmental Biology, Vanderbilt University School of Medicine, PMB 407935, 465 21st Ave. S, Nashville, TN 37232, USA; Department of Cell and Developmental Biology, Vanderbilt University School of Medicine, PMB 407935, 465 21st Ave. S, Nashville, TN 37232, USA; Department of Cell and Developmental Biology, Vanderbilt University School of Medicine, PMB 407935, 465 21st Ave. S, Nashville, TN 37232, USA; Department of Cell and Developmental Biology, Vanderbilt University School of Medicine, PMB 407935, 465 21st Ave. S, Nashville, TN 37232, USA; Department of Cell and Developmental Biology, Vanderbilt University School of Medicine, PMB 407935, 465 21st Ave. S, Nashville, TN 37232, USA; Department of Cell and Developmental Biology, Vanderbilt University School of Medicine, PMB 407935, 465 21st Ave. S, Nashville, TN 37232, USA; Department of Cell and Developmental Biology, Vanderbilt University School of Medicine, PMB 407935, 465 21st Ave. S, Nashville, TN 37232, USA

**Keywords:** spindle pole body, *Schizosaccharomyces pombe*, Ppc89, septation initiation network, fission yeast, cytokinesis

## Abstract

Centrosomes and spindle pole bodies (SPBs) are important for mitotic spindle formation and also serve as signaling platforms. In the fission yeast *Schizosaccharomyces pombe*, genetic ablation and high-resolution imaging indicate that the α-helical Ppc89 is central to SPB structure and function. Here, we developed and characterized conditional and truncation mutants of *ppc89*. Alleles with mutations in 2 predicted α-helices near the C-terminus were specifically defective in anchoring Sid4, the scaffold for the septation initiation network (SIN), and proteins dependent on Sid4 (Cdc11, Dma1, Mto1, and Mto2). Artificial tethering of Sid4 to the SPB fully rescued these *ppc89* mutants. Another *ppc89* allele had mutations located throughout the coding region. While this mutant was also defective in Sid4 anchoring, it displayed additional defects including fragmented SPBs and forming and constricting a second cytokinetic ring in 1 daughter cell. These defects were shared with a *ppc89* allele truncated of the most C-terminal predicted α-helices that is still able to recruit Sid4 and the SIN. We conclude that Ppc89 not only tethers the SIN to the SPB but is also necessary for the integrity of the SPB and faithful coordination of cytokinesis with mitosis.

## Introduction

Centrosomes are membrane-less organelles that serve as major microtubule organizing centers (MTOCs) in human cells (reviewed in Bornens 2021; Vasquez-Limeta and Loncarek 2021). Because they form poles of the mitotic spindle, centrosome amplification or structural defects can lead to errors in chromosome segregation and are frequently observed in cancer (Ganem *et al.* 2007; Godinho *et al.* 2014; Ryniawec and Rogers 2021). Centrosomes also serve as platforms upon which signaling hubs are established to regulate multiple other processes including the cell cycle, cell polarity, asymmetric cell division, cellular responses to DNA damage, sensory perception, and cellular aging (reviewed in Chavali *et al.* 2014; Bornens 2021; Langlois-Lemay and D'Amours 2022; Lin *et al.* 2022). Although structurally distinct from centrosomes, the *Saccharomyces cerevisiae* and *Schizosaccharomyces pombe* spindle pole bodies (SPBs) share with centrosomes important biological roles, composition, and regulation and studies of SPB organization, functions, and mechanisms of duplication have provided many insights into centrosome biology (Kilmartin 2014; Fu *et al.* 2015; Cavanaugh and Jaspersen 2017; Ito and Bettencourt-Dias 2018; Jaspersen 2021).

The architecture of the *S. cerevisiae* SPB is well-established through a series of super-resolution imaging, immuno-electron microscopy, in vitro reconstitution, and molecular modeling studies (reviewed in Ito and Bettencourt-Dias 2018; Jaspersen 2021). Embedded in the nuclear envelope (NE) throughout the cell cycle, its ∼18 components are organized into distinct layers around a hexagonal lattice core of Spc42 (Bullitt *et al.* 1997; Viswanath *et al.* 2017). Together with another core SPB component, Spc29, Spc42 attaches to Sfi1 and centrin Cdc31 (Ruthnick *et al.* 2021). Sfi1 and Cdc31 form the half-bridge, an appendage which mediates new SPB formation and then becomes the full bridge linking the old and new SPBs until their separation during mitosis (Kilmartin 2003; Li *et al.* 2006; Ruthnick and Schiebel 2016).

The precise organization of the *S. pombe* SPB is less clear (reviewed in Jaspersen 2021). It is not distinctly layered when viewed by electron microscopy at any stage of the cell cycle (Ding *et al.* 1997; Uzawa *et al.* 2004). Also, *S. pombe* SPBs migrate in and out of the NE during the mitotic cell cycle (Ding *et al.* 1997; Uzawa *et al.* 2004). The mother SPB, which serves as an MTOC for interphase and astral microtubules, is bound to the cytoplasmic face of the NE by an undefined tether where it is duplicated similarly to the *S. cerevisiae* SPB (Bouhlel *et al.* 2015). At mitosis, the linked old and new SPBs embed into the NE where they serve as poles for the intranuclear mitotic spindle. The 2 SPBs separate during prometaphase and at the end of mitosis, *S. pombe* SPBs once again move out of the NE (Ding *et al.* 1997; Uzawa *et al.* 2004). These SPB movements are accompanied by redistributions of certain SPB components into and out of SPB-encircling rings (Bestul *et al.* 2021).

Though distinct in morphology, many structural *S. pombe* SPB components are related to those in *S. cerevisiae*; these include the bridge components Sfi1 and centrin Cdc31 (Kilmartin 2003; Paoletti *et al.* 2003; Lee *et al.* 2014; Bouhlel *et al.* 2015), pericentrin-like Pcp1 (Flory *et al.* 2002; Fong *et al.* 2010), microtubule nucleating factors (reviewed in Lin *et al.* 2015), and signaling proteins including the Polo-like kinase Plo1 (Bahler, Steever, *et al.* 1998; Mulvihill *et al.* 1999), CK1 kinases (Johnson *et al.* 2013), Cdk1–Cdc13 cyclin (Alfa *et al.* 1990; Decottignies *et al.* 2001), multiple phosphatases (e.g. Clp1 and SIP complex; Cueille *et al.* 2001; Trautmann *et al.* 2001; Singh *et al.* 2011), and all members of the septation initiation network (SIN; Morrell, Tomlin, *et al.* 2004), a GTPase-driven protein kinase cascade required for cell division (Simanis 2015; Cullati and Gould 2019). The α-helical protein Ppc89 forms the structural core of the *S. pombe* SPB, is the first to join Sfi1 at the new SPB, and is considered to be a functional analog of *S. cerevisiae* Spc42 (Rosenberg *et al.* 2006; Bestul *et al.* 2017). That there is no analog of Spc29 in *S. pombe* led to the hypothesis that Spc42 and Spc29 may have arisen during evolution by gene splitting of an ancestral *ppc89* (Ito and Bettencourt-Dias 2018; Jaspersen 2021). Overproduction of Ppc89 results in massive SPB enlargement, accumulation of other SPB proteins into the enlarged structures, and cell death (Rosenberg *et al.* 2006; Chen *et al.* 2024). Reciprocally, repression of *ppc89* expression causes loss of multiple proteins from the SPB and this also results in cell death (Rosenberg *et al.* 2006).

Super-resolution and immuno-electron microscopy imaging indicate that Ppc89 is positioned perpendicularly relative to the NE with its N-terminus proximal to the NE and its C-terminus extending away (Bestul *et al.* 2017). In accord, Ppc89 interacts with the SIN SPB anchor, Sid4 (Chang and Gould 2000; Rosenberg *et al.* 2006) near its C-terminus and the pericentrin Pcp1 (Flory *et al.* 2002) closer to its N-terminus (Chen *et al.* 2024) but it is not known how or if other key SPB proteins physically or functionally interact with Ppc89, how Ppc89 is tethered to the NE, or how Ppc89 organizes mitotic signaling modules at the SPB.

To help fill these gaps in understanding, we developed temperature-sensitive (ts) and truncation mutants of *ppc89*. Using these new tools, we found that the localization of Sid4 and all SPB-localized SIN components, as well as that of the cytoplasmic γ-tubulin complex tether Mto1–Mto2 (Sawin *et al.* 2004; Venkatram *et al.* 2004; Samejima *et al.* 2010; Lynch *et al.* 2014), depend on Sid4 interaction with 2 predicted Ppc89 α-helices (numbers 6 and 7). These results further indicate that cytoplasmic microtubule nucleation and SIN signaling may be regulated coordinately due to their proximity. Additionally, we found that Ppc89 could be truncated of its most C-terminal predicted α-helices (numbers 8 and 9) without loss of Sid4 or complete loss of viability. However, loss of α-helices 8 and 9 led to temperature-dependent growth, the accumulation of fragmented Ppc89 foci during mitosis, and the appearance of uninucleate cells with an off-center septum due to the formation and constriction of a second cytokinetic ring in 1 daughter cell soon after its birth. These results suggest that Ppc89 not only scaffolds SIN components but has a separable role in ensuring SPB integrity and that cytokinesis occurs only once per cell cycle.

## Materials and Methods

### Yeast methods

*Schizosaccharomyces pombe* strains used in this study (Supplementary Table 1) were grown in yeast extract (YE; Forsburg and Rhind 2006). For serial dilution growth assay, cells were grown in liquid YE at 25°C. Three 10-fold serial dilutions starting at 4 × 10^6^ cells/mL of each strain were made and 3 μL of each dilution were spotted onto YE agar plates. Strain construction was accomplished through tetrad analysis using standard methods (Moreno *et al.* 1991). Cells were fixed with 70% ethanol for DAPI and methyl blue (MB) staining as described previously (Roberts-Galbraith *et al.* 2009).

Tagged strains were generated by endogenously tagging the 3′ end of *ppc89* with sequences encoding *mNG:hphMX6, mCherry:natMX6*, and *GFP:hphMX6* using pFA6 cassettes, as previously described (Bahler, Wu, *et al.* 1998) and lithium acetate transformations (Keeney and Boeke 1994). G418 (100 mg/mL; Sigma-Aldrich), Hygromycin B (50 mg/mL; Thermo Fisher), or nourseothiricin (100 µg/mL; Sigma-Aldrich) was used for the selection of *kanMX6*, *hphMX6*, or *natMX6* cells, respectively. Tagged strains were confirmed by whole-cell PCR. All fusion proteins were expressed from their native promoters at their chromosomal loci.

The yeast 2-hybrid system was used in this study as described previously (James *et al.* 1996). Various portions of *ppc89^+^* or *sid4*^+^ were amplified by PCR from genomic DNA and cloned into the bait plasmid pGBT9 and/or the prey plasmid pGAD424 (Clontech). To test for protein interactions, both bait and prey plasmids were cotransformed into *S. cerevisiae* strain PJ69-4A. Leu^+^ and Trp^+^ transformants were selected and then scored for positive interactions by streaking onto synthetic dextrose plates lacking histidine and synthetic dextrose plates lacking adenine and histidine.

Truncation of *ppc89* and *ppc89-L756P,I770V* were generated by PCR-mediated insertions of epitope tags after amino acid 707 or with relevant mutations before the stop codon, respectively (Bahler, Steever, *et al.* 1998).

### Isolation of temperature-sensitive alleles with error-prone PCR

ts alleles of *ppc89* were constructed based on the previously described protocol (Tang *et al.* 2019) but used EX Taq polymerase (Takara, 4025) and accompanying dNTPs (Takara, RR01BM).

### Microscopy

Strains for live-cell imaging experiments were grown at 25°C in YE and then shifted to 36°C for 3 h. Live-cell images were acquired using a Zeiss Axio Observer inverted epifluorescence microscope with a 63× oil objective (1.46 NA) and captured using Zeiss ZEN 3.0 (Blue edition) software and an Axiocam 503 monochrome camera. Images were acquired with a z-stack step size of 0.5 µm and a total of 10 z-slices. Images included in the figures were not deconvolved and were max projected. For DAPI/MB images, a singular medial z-slice was obtained. All images were further processed using ImageJ (Schindelin *et al.* 2012).

**Fig. 1.**
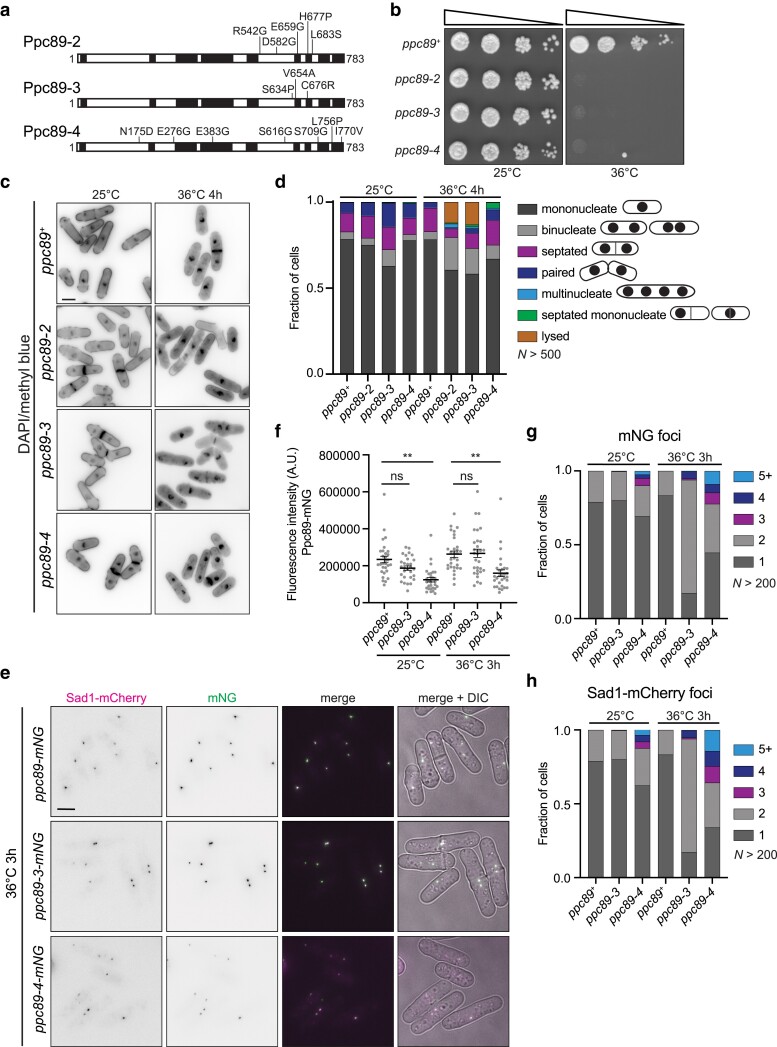
Characterization of the *ppc89* ts alleles. a) Schematics, drawn to scale, of Ppc89 and the AlphaFold predicted α-helices in black. The mutations encoded by the ts alleles are indicated. b) Ten-fold serial dilutions of the indicated strains grown at the indicated temperatures on YE agar for 2–3 days. c) Fixed-cell imaging of the indicated strains grown at 25°C and shifted to 36°C for 4 h. Samples were collected at both time points and stained with DAPI and MB. d) Quantification of the phenotypes from (c). *N* > 500 cells for each condition and strain from 3 independent experiments. e) Live-cell imaging of cells expressing Ppc89-mNG, Ppc89-3-mNG, or Ppc89-4-mNG with Sad1-mCherry. Cells were grown at 25°C and were shifted to 36°C for 3 h and imaged at both time points. f) Quantification of mNG SPB fluorescence intensity from (e). *N* ≥ 29 cells for each condition and strain from 3 independent experiments. Error bars represent mean ± SEM. *P*-values were obtained with a 1-way ANOVA with Tukey's post hoc test. *ppc89-mNG* versus *ppc89-3-mNG* at 25°C, *P* = 0.38*. ppc89-mNG* versus *ppc89-3-mNG* at 36°C, *P* > 0.9999. *ppc89-mNG* versus *ppc89-4-mNG* at 25°C and 36°C, **P ≤ 0.01. ns, no significance. (g, h) Quantification of Ppc89-mNG and Sad1-mCherry foci per cell from (e). *N* > 200 cells for each condition and strain from 3 independent experiments. Scale bar, 5 µm.

**Fig. 2.**
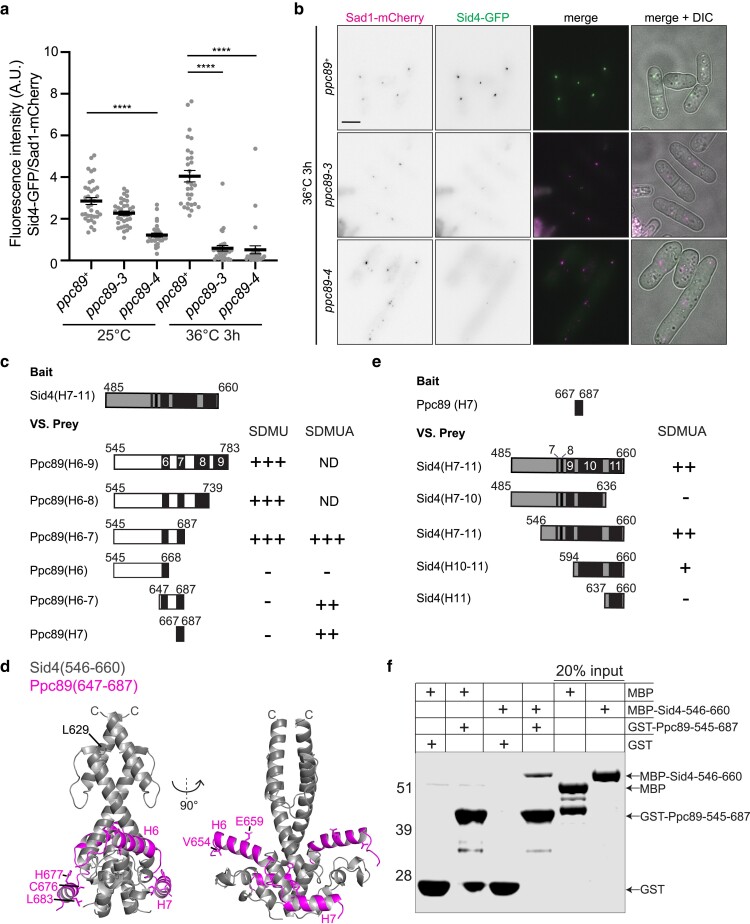
Live-cell imaging of cells expressing Sid4-GFP and Sad1-mCherry in *ppc89*^+^, *ppc89-3*, or *ppc89-4* cells. Cells were grown at 25°C and were shifted to 36°C for 3 h and images were acquired at both time points. Scale bar, 5 µm. a) Quantification of SPB fluorescence intensity from cells as in (b) and S2B. Sid4-GFP SPB fluorescence intensity was divided by Sad1-mCherry SPB fluorescence intensity. *N* ≥ 30 cells for each condition and strain from 3 independent experiments. From 3 independent experiments. Error bars represent mean ± SEM. *P*-values were obtained with a 1-way ANOVA with Tukey's post hoc test. ****P ≤ 0.0001. b) Representative images from the indicated strains. (c, e) Yeast 2-hybrid analysis was performed with *S. cerevisiae* strain PJ69-4A which was cotransformed with plasmids expressing the indicated Ppc89 and Sid4 fragments. Black boxes indicate regions of predicted α-helices. *leu*^+^  *trp*^+^ transformants were tested for growth on −his and -ade plates (SDMU) or -his plates (SDMUA). “+++” indicates strong growth, “++” indicates little growth, and “−” indicates no growth. d) AlphaFold3 predicted structure of the Ppc89-Sid4 complex. Two copies of Ppc89s residues 647-687 were modeled with 2 copies of Sid4 residues 546-660. Ppc89 is in magenta and Sid4 is in gray. f) In vitro binding assay with bead-bound GST or GST-Ppc89(545-687) and soluble MBP or MBP-Sid4(546-660). Samples were washed, resolved by SDS-PAGE, and stained with Coomassie blue.

**Fig. 3.**
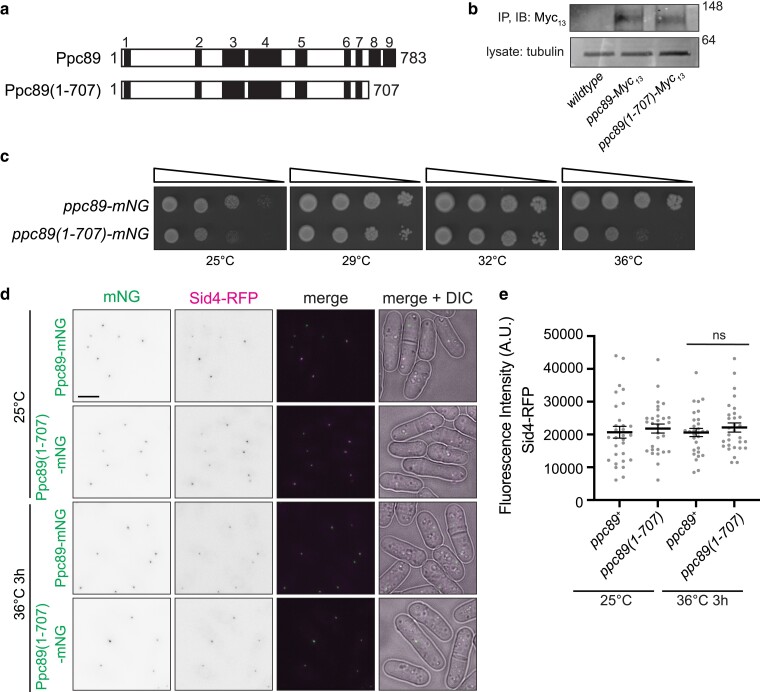
Ppc89 α-helices 8 and 9 are dispensable for promoting Sid4 localization. a) Schematics, drawn to scale, of Ppc89 and Ppc89(1-707) with the AF-predicted α-helices in black and numbered above. b) Ppc89-Myc13 or Ppc89(1-707)-Myc13 was immunoprecipitated from cell lysates with anti-Myc (9E10) antibody conjugated to protein G beads and incubated at 4°C for 1 h. Bound protein was resolved by SDS-PAGE and visualized by Western blot. The blot shown is representative of 2 independent replicates. c) Ten-fold serial dilutions of the indicated strains grown at the indicated temperatures on YE agar for 2-3 days. d) Live-cell imaging of cells expressing Ppc89-mNG or Ppc89(1-707)-mNG and Sid4-RFP. Cells were grown at 25°C and were shifted to 36°C for 3 h. Images were acquired at both time points. Scale bar, 5 µm. e) Quantification of RFP SPB fluorescence intensity of strains from (d). *N* = 30 cells for each condition and strain from 3 independent experiments. Error bars represent mean ± SEM. *P* = 0.88; 1-way ANOVA with Tukey's post hoc test. ns, no significance.

**Fig. 4.**
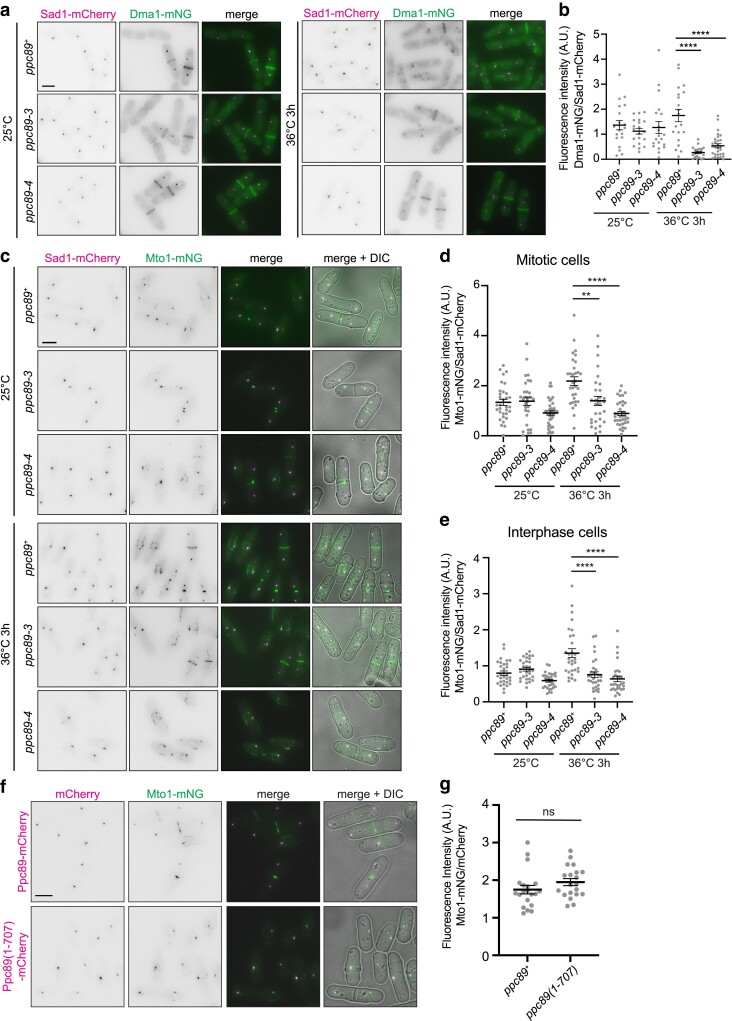
*ppc89-3* and *ppc89-4* have reduced Dma1 and Mto1 SPB localization. a) Live-cell imaging of cells expressing Dma1-mNG Sad1-mCherry in *ppc89^+^*, *ppc89-3*, or *ppc89-4* background. Cells were grown at 25°C and were shifted to 36°C for 3 h. Images were acquired at both time points. b) Quantification of SPB fluorescence intensity in septated cells from (a). Dma1-mNG SPB fluorescence intensity was divided by Sad1-mCherry SPB fluorescence intensity. *N* ≥ 20 cells for each condition and strain from 2 independent experiments. Error bars represent mean ± SEM. *P-*values were determined with a 1-way ANOVA with Tukey's post hoc test. ****P ≤ 0.0001. c) Live-cell imaging of cells expressing Mto1-mNG Sad1-mCherry in *ppc89^+^*, *ppc89-3*, or *ppc89-4* background. Cells were grown at 25°C and were shifted to 36°C for 3 h. Images were acquired at both time points. d) Quantification of SPB fluorescence intensity of mitotic cells from (c). *N* ≥ 30 cells for each condition and strain from 3 independent experiments. e) Quantification of SPB fluorescence intensity of interphase cells from (c). Mto1-mNG SPB fluorescence intensity was divided by Sad1-mCherry SPB fluorescence intensity. *N* ≥ 30 cells for each condition and strain from 3 independent experiments. Error bars represent mean ± SEM. One-way ANOVA with Tukey's post hoc test; **P ≤ 0.01, ****P ≤ 0.0001. f) Live-cell imaging of cells expressing Ppc89-mCherry or Ppc89(1–707)-mCherry and Mto1-mNG. Cells were grown at 25°C and were shifted to 36°C for 3 h. Representative images are shown for cells after shift to 36°C. Scale bar, 5 µm. g) Quantification of mNG SPB fluorescence intensity divided by mCherry SPB fluorescence intensity of strains from (a). *N* = 20 cells for each condition and strain from 2 independent experiments. Error bars represent mean ± SEM. *P* = 0.18; unpaired, 2-tailed Student's *t*-test. ns, no significance. Scale bar, 5 µm.

Images for quantification of SPB intensity were not deconvolved and were sum projected. Intensity measurements with a region of interest drawn around the SPB were made with ImageJ. For Figs. 1, 3, and 4, the camera background was subtracted from intensity measurements, for Figs. 2, 4b, and Supplementary Figs. 2 and 3, cytoplasmic background intensity was subtracted from intensity measurements, and in Fig. 4d and e, nuclear background was subtracted from intensity measurements (Waters 2009).

Time-lapse imaging was performed using a Leica Thunder imager system including a DMI8 inverted microscope, 63X plan apo oil objective (1.40 NA), a Leica K8 sCMOS camera, standard excitation and emission filters, and an LED light source (Leica Microsystems). Images were acquired using Leica Application Suite X (LAS X) software (Leica Microsystems). A CellASIC ONIX microfluidics perfusion system (Millipore Sigma) was used, and cells were loaded into Y04C plates for 10 s at 8 psi. YE liquid medium flowed through the chamber at 5 psi throughout imaging. Z-series optical sections were taken at 0.5 µm spacing and images were acquired every 2 min. Five movies of mutant cells were taken and 2 movies of wildtype cells were taken for comparison.

### Quantification and statistical analyses

Calculations of the mean, standard error of the mean (SEM), and statistical significance were performed with Prism 8.0 (GraphPad Software). Significance was defined by a *P-*value equal to or less than 0.05. One-way ANOVA was used with Tukey's post hoc test for multiple comparisons. Student's *t*-test in Fig. 4g was unpaired and 2-tailed.

### Recombinant protein expression

MBP-Sid4 and GST-Ppc89 fragments were produced in Rosetta2(DE3)pLysS bacteria grown in Terrific broth supplemented with 100 μg/mL ampicillin and 34 μg/mL chloramphenicol by incubating on ice for 15 min, adding 0.4 mM isopropyl β-D-1-thiogalactopyranoside (IPTG; Fisher Scientific; BP1755), and incubating the cells for 16–18 h at 18°C.

MBP and MBP-Sid4(546–660) were purified on amylose resin (New England Biolabs) in 20 mM Tris-HCl pH 7.4, 150 mM NaCl, 1 mM ethylenediaminetetraacetic acid (EDTA), 0.1% NP40, 1 mM dithiothreitol (DTT), 1 mM phenylmethanesulfonyl fluoride (PMSF), 1.3 mM benzamidine, and protease inhibitor cocktail (Roche). GST and GST-Ppc89 were purified with GST-bind resin (Millipore Sigma) in 4.3 mM sodium phosphate pH 7.3, 137 mM NaCl, 2.7 mM KCl, 1 mM DTT, 0.1% NP40, 1 mM PMSF, 1.3 mM benzamidine, and protease inhibitor cocktail. GST and GST-Ppc89 were eluted with glutathione (50 mM Tris-HCl pH 8.0, 10 mM glutathione).

### In vitro binding assay

Ten micrograms recombinant GST or GST-Ppc89 protein bound to GST beads were incubated in 100 µL 1× GST-bind buffer (4.3 mM sodium phosphate, pH 7.3, 137 mM NaCl, 2.7 mM KCl, 1 mM DTT, 0.1% NP-40) containing 5% Bovine Serum Albumin for 30 min at 4°C to prevent nonspecific binding. Next, recombinant 10 µg MBP or MBP-Sid4 proteins were added and incubated with the beads for an additional 30 min at 4°C. The beads were first washed 3 times with 1 mL 1× GST-bind buffer and then washed an additional 3 times with 1 mL GST-bind buffer containing 300 mM NaCl to remove unbound proteins. Bead-bound proteins were resuspended in 20 µL 2× SDS sample buffer, heated at 95°C for 5 min, and separated on a 4–12 NuPAGE % Bis-Tris gel with MOPS buffer (NP0322BOX, Invitrogen). Proteins were visualized by Coomassie blue staining.

### AlphaFold3 structure predictions

Protein structure predictions were generated with the AlphaFold3 (AF) server (Abramson *et al.* 2024) and visualized using the PyMOL molecular graphics system version 3.0 Schrodinger, LLC.

### Immunoprecipitations and immunoblotting

Cells at an optical density (OD)595 of ≤0.5 were collected by centrifugation in 50 mL tubes. For each immunoprecipitation, a 30 OD pellet was collected, resuspended in NP-40 buffer [6 mM Na_2_HPO_4_, 4 mM NaH_2_PO_4_, 1% NP-40, 150 mM NaCl, 50 mM NaF, 0.1 mM Na_3_VO_4_, 2 mM EDTA, 1 mM PMSF, 2 mM benzamidine, 0.5 mM diisopropyl fluorophosphate (DIFP), 5 μg/mL leupeptin] and protease inhibitor tablet (cOmplete Protease Inhibitor Cocktail, Roche), transferred to a 15 mL tube and pelleted by centrifugation. Whole-cell lysates were then prepared in NP-40 buffer as previously described (Gould *et al.* 1991). Proteins were immunoprecipitated from protein lysates using anti-Myc 9E10 (Vanderbilt Antibody and Protein Resource Core) at 2 µg/mL followed by Protein G Sepharose beads (GE Healthcare).

For immunoblotting, proteins were resolved by 8% or 10% Tris-glycine gels, transferred by electroblotting to a polyvinylidene difluoride (PVDF) membrane (Immobilon FL; Millipore Sigma) and incubated with the specified primary antibodies at 1 µg/mL. Primary antibodies were detected with secondary antibodies coupled to IRDye680 or IRDye800 (LI-COR Biosciences) and visualized using an Odyssey Imaging System (LI-COR Biosciences).

## Results

### Isolation and characterization of *pcp89* mutants

Three ts alleles of *ppc89^+^* were produced by random mutagenesis of *ppc89^+^* (Tang *et al.* 2019). *ppc89-2*, *ppc89-3*, and *ppc89-4* each contain a unique set of mutations (Fig. 1a). The mutations within *ppc89-2* and *ppc89-3* are confined to the C-terminal region of the open reading frame (ORF), while *ppc89-4* has mutations distributed throughout the entire ORF (Fig. 1a). *ppc89-2*, *ppc89-3*, and *ppc89-4* grow similarly to wildtype at 25°C but do not form colonies at 36°C (Fig. 1b). Wildtype cells at both temperatures and all 3 *ppc89* mutants at 25°C showed the expected proportion of mononucleate, binucleate, septated, and paired cells (Fig. 1c and d). At 36°C, *ppc89-2* and *ppc89-3* displayed an increased proportion of binucleate cells with “kissing” nuclei in which nuclei return to the cell center after a failed cytokinesis, as well as cell lysis (Fig. 1c and d), phenotypes reminiscent of SIN mutants (Simanis 2015). A portion of *ppc89-4* cells at 36°C showed an additional abnormal phenotype—cells with a septum but only 1 nucleus (Fig. 1c and d). Because of the similar region of mutations and the indistinguishable phenotypes of *ppc89-2* and *ppc89-3*, we only characterized only *ppc89-3* and *ppc89-4* further.

To determine if the mutations affected Ppc89 localization, C-terminal tags were introduced to Ppc89, Ppc89-3, and Ppc89-4 by appending sequences encoding mNeonGreen (mNG) at the endogenous loci in strains that also expressed Sad1-mCherry as a SPB marker (Hagan and Yanagida 1995). Ppc89-3-mNG localized to the SPB at both permissive and restrictive temperatures similar to Ppc89-mNG (Fig. 1e and f and Supplementary Fig. 1a). In contrast, Ppc89-4-mNG showed a ∼50% reduction in SPB fluorescence intensity at both temperatures compared to wildtype (Fig. 1e and f and Supplementary Fig. 1a). In *ppc89-3* cells at the restrictive temperature, we noticed an increased proportion of cells displaying 2 or 4 Ppc89-3-mNG and Sad1-mCherry foci, indicative of cytokinesis failure (Fig. 1g and h). In contrast, *ppc89-4* cells displayed 3, 4, or ≥5 Ppc89-4-mNG and Sad1-mCherry foci (Fig. 1g and h), indicating that the integrity of the SPB as a whole is disrupted in *ppc89-4* cells whereas it appears to remain intact in *ppc89-3* cells.

### Ppc89 mutants impact Ppc89–Sid4 interaction

Because the *ppc89-3* phenotypes resembled SIN mutant phenotypes, we screened for genetic interactions with established alleles defective in SIN signaling (Bahler, Steever, *et al.* 1998; Balasubramanian *et al.* 1998; Salimova *et al.* 2000). We found that *ppc89-3* was synthetically lethal with *sid4-SA1* and synthetically sick with *spg1-106*, *mob1-R4*, and *plo1-25* (Supplementary Fig. 1b and c). *ppc89-4* was also synthetically lethal with *sid4-SA1* (Supplementary Fig. 1c). These results, combined with the fact that the *ppc89* alleles contain mutations within the C-terminal region that binds Sid4 (Rosenberg *et al.* 2006) led us to investigate Sid4-GFP localization. At 25°C, Sid4-GFP SPB intensity was reduced by ∼30% in *ppc89-3* and 60% in *ppc89-4* compared to wildtype (Fig. 2a and Supplementary Fig. 2a). At 36°C, Sid4-GFP SPB intensity was further reduced by ∼90% in *ppc89-3* and *ppc89-4* compared to wildtype (Fig. 2a and b). These results indicate that the Sid4-Ppc89 interaction is disrupted in both *ppc89-3* and *ppc89-4* cells at restrictive temperature.

It was previously shown that a fragment of Ppc89 predicted to contain a linker region and α-helices 6–9 interacted with a C-terminal Sid4 fragment in a yeast 2-hybrid assay (Rosenberg *et al.* 2006). We reasoned that the location of the mutations in *ppc89-3* and *ppc89-4* would help to better define the region within Ppc89 that mediates Sid4 binding. Ppc89-3 contains a V654A mutation within α-helix 6 and a C676R mutation within α-helix 7 (Fig. 1a). In accord with these locales, a smaller fragment of Ppc89 containing the linker region, α-helix 6, and α-helix 7 robustly interacted with this Sid4 fragment in a 2-hybrid assay (Fig. 2c). Under less stringent selection conditions, a Ppc89 fragment containing α-helix 6 and 7 or only α-helix 7 also interacted with Sid4 (Fig. 2c). Although *ppc89-4* does not have mutations within α-helix 6 or α-helix 7, it does contain a mutation within the linker region upstream of α-helix 6 (S616G; Fig. 1a). We conclude from these results that Sid4 interaction likely involves Ppc89 α-helices 6 and 7 as well as a linker region and that α-helices 8 and 9 at the C-terminus of Ppc89 are not required.

To gain further insight into how these 2 proteins may bind, we interrogated the interaction with AF (Abramson *et al.* 2024). Sid4 is a dimeric protein of 660 aa (Chang and Gould 2000) and AF predicts it contains eleven α-helices (Jumper *et al.* 2021; Varadi *et al.* 2022). A fragment of Sid4 containing predicted α-helices 7–11 localizes to the SPB and interacts with Ppc89 (Chang and Gould 2000). In the AF-generated model, 2 molecules of Ppc89 α-helices 6 and 7 bound either side of a dimer of Sid4 α-helices 7–11 (Fig. 2d; Mirdita *et al.* 2022). The mutations encoded by the *ppc89-2* and *ppc89-3* were mapped in proximity to the Ppc89–Sid4 interface (Fig. 2d). The single-site mutation in *sid4-SA1* encodes an L629P substitution within α-helix 11 and the protein encoded by this allele does not localize to the SPB at the restrictive temperature (Chang and Gould 2000). The L629P mutation maps within an area that may be important for Sid4 self-dimerization which may be a prerequisite for Ppc89 binding (Chang and Gould 2000; Fig. 3d). Consistent with the AF model, truncation of the Sid4 α-helix 11 resulted in no interaction with Ppc89 but α-helix 11 alone was not sufficient for interaction (Fig. 2e). A Sid4 fragment containing α-helices 10 and 11 showed a weak interaction (Fig. 2e). Also, as expected, removal of the unstructured region upstream of Sid4 α-helix 7 had no impact on Ppc89 binding in the 2-hybrid assay (Fig. 2e). A direct Ppc89–Sid4 interaction was confirmed in vitro with recombinantly produced GST-Ppc89(545–687) and MBP-Sid4(546–660) (Fig. 2f). Overall, our data indicate that Ppc89 α-helices 6 and 7 directly bind an interface of a Sid4 dimer formed by helices 7–11.

### Ppc89 C-terminal residues are dispensable for viability and Sid4 interaction

To verify that predicted Ppc89 α-helices 8 and 9 are not required to support Sid4 interaction, we generated a C-terminal truncation mutant by introducing sequences encoding mNG or Myc_13_ after the codon specifying amino acid 707 at the endogenous *ppc89* locus (Fig. 3a). An immunoblot confirmed that the insertion of sequences after residue 707 resulted in a truncated protein (Fig. 3b). *ppc89(1–707)-mNG* cells were viable but ts (Fig. 3c). Both Ppc89(1–707)-mNG and Sid4-RFP localized to the SPB with comparable intensity to wildtype at both 25°C and 36°C (Fig. 3d and e). Thus, Ppc89 α-helices 8 and 9 are not necessary for the Ppc89–Sid4 interaction.

### Ppc89 mutants affect the localization of other SPB proteins

Centriolin-like Cdc11, the ubiquitin ligase Dma1, and the γ-tubulin complex linker Mto1 all depend on Sid4 for their SPB localization (Krapp *et al.* 2001; Guertin *et al.* 2002; Tomlin *et al.* 2002; Samejima *et al.* 2010). Sid4 recruits Cdc11 to the SPB to scaffold other components of the SIN (Krapp *et al.* 2001; Tomlin *et al.* 2002). Dma1 also directly binds and ubiquitylates Sid4 to regulate SIN signaling, and both Cdc11 and Dma1 are completely dependent on Sid4 for SPB localization (Guertin *et al.* 2002; Tomlin *et al.* 2002). Mto1 recruits the γ-tubulin complex for nucleation of interphase and astral mitotic microtubules, and it depends on Cdc11 and Sid4 for SPB localization, although it is unclear how exactly Mto1 links into the SPB (Sawin *et al.* 2004; Venkatram *et al.* 2004; Samejima *et al.* 2010; Lynch *et al.* 2014). As expected, Dma1-mNG, Cdc11-GFP, and Mto1-mNG were lost from SPBs in *ppc89-3* and *ppc89-4* cells at restrictive temperature, but Mto1-mNG localized normally to SPBs in *ppc89(1–707)* cells (Fig. 4a–g; Supplementary Fig. 2b and c). Mto2 requires Mto1 for SPB localization (Janson *et al.* 2005; Samejima *et al.* 2005; Venkatram *et al.* 2005), and we also observed a reduction in Mto2-mNG SPB signal at 36°C in *ppc89-3* and *ppc89-4* compared to wildtype (Supplementary Fig. 2d and e).

The localizations of the half-bridge protein Sfi1 and Pcp1 were also examined in *ppc89-3* and/or *ppc89-4* cells relative to Sad1-mCherry signal. Neither Sfi1-mCherry nor Sad1-mCherry signal was reduced in *ppc89-3* or *ppc89-4* cells (Supplementary Fig. 3a and b). However, matching the 50% loss of Ppc89-4 itself (Fig. 1e and f), Pcp1 showed ∼50% reduction in *pcp89-4* cells (Supplementary Fig. 3c), consistent with Pcp1 binding Ppc89 for its SPB localization (Chen *et al.* 2024).

### *ppc89-3* and *ppc89-4* are defective in Sid4 anchoring

**Fig. 5.**
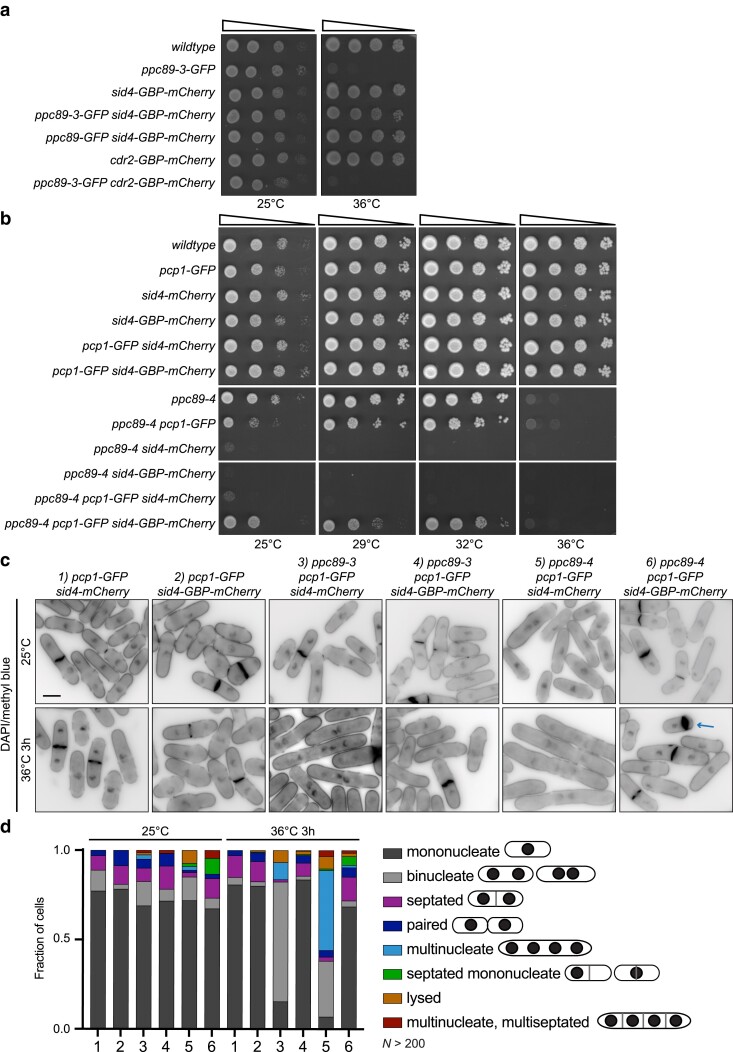
Artificially targeting Sid4 to the SPB fully rescues *ppc89-3* growth defect. (a, b) Ten-fold serial dilutions of the indicated strains grown at the indicated temperatures on YE agar for 2–3 days. c) Fixed-cell imaging of the indicated strains grown at 25°C and shifted to 36°C for 3 h. Samples were collected at both time points and stained with DAPI and MB. Scale bar, 5 µm. d) Quantification of the phenotypes from (c). *N* > 200 cells for each condition and strain from 3 independent experiments.

The above results led us to ask whether *ppc89-3* and *ppc89-4* cells are solely defective in Ppc89–Sid4 interaction. To test this, we tagged *ppc89-3* with sequences encoding GFP and we combined this allele with *sid4^+^* tagged with sequences encoding GFP-binding protein (GBP)-mCherry so that Sid4 could be artificially tethered to the SPB (Rothbauer *et al.* 2006, 2008). Because Sid4 is a stable SPB component (Morrell, Tomlin, *et al.* 2004), we reasoned that tethering it to the SPB was unlikely to interfere with its function. Supporting the idea that *ppc89-3* cells are specifically defective in Sid4 binding, *ppc89-3-GFP sid4-GBP-mCherry* cells were able to grow at 36°C, and tethering the unrelated cortical node protein Cdr2 (Morrell, Nichols, *et al.* 2004) to Ppc89 did not rescue growth of *ppc89-3-GFP* (Fig. 5a).

We were unable to tag Ppc89-4 with GFP so we tested whether tethering Sid4 to Pcp1-GFP would rescue *ppc89-3* and if so, *ppc89-4* as well. *ppc89-3* was rescued when Sid4 was tethered to Pcp1-GFP, as it had been when tethered to Ppc89-3 directly (Supplementary Fig. 3d). Imaging confirmed that Sid4-GBP-mCherry but not Sid4-mCherry was present at the SPB in *ppc89-3 pcp1-GFP* cells at 36°C (Supplementary Fig. 4a). Sid4 tethered to the SPB via Pcp1-GFP did not rescue growth of *ppc89-4* cells at 36°C but the *ppc89-4 pcp1-GFP sid4-mCherry* strain grew at 32°C, and imaging confirmed that Sid4-GBP-mCherry but not Sid4-mCherry was present at the SPB at 36°C (Fig. 5b and Supplementary Fig. 4a). These results suggested that the lethality of *ppc89-4* is only partially due to defective Sid4-Ppc89 interaction and SIN failure.

To better understand Sid4-independent defects in *ppc89-4*, we examined the phenotypes of the strains. At 36°C, both *ppc89-3* and *ppc89-4* cells with *pcp1-GFP sid4-mCherry* were multinucleated, indicative of cytokinesis failure (Fig. 5c, third and fifth bottom panels from left). The addition of the tagged *sid4* and *pcp1* alleles exacerbated the cell growth (Fig. 5b and Supplementary Fig. 3d) and cell division defects of the *ppc89* ts alleles (Figs. 1c, d and 5c, d). The phenotypes of *ppc89-3* cells with Sid4 tethered to the SPB were similar to the wildtype control (Fig. 5c, fourth bottom panel from left). In contrast, *ppc89-4* cells with Sid4 tethered to the SPB displayed a significant percentage of uninucleate cells with an off-center septum and also multiple Ppc89-4 foci (Fig. 5c and d and Supplementary Fig. 4). These results underscore that (i) *ppc89-3* is specifically defective in Sid4 anchoring, (ii) the cytokinesis failure observed in *ppc89-4* cells is due to loss of SPB Sid4, and (iii) the mononucleate septated cell and multiple SPB foci phenotypes of *ppc89-4* are independent of Sid4 function.

**Fig. 6.**
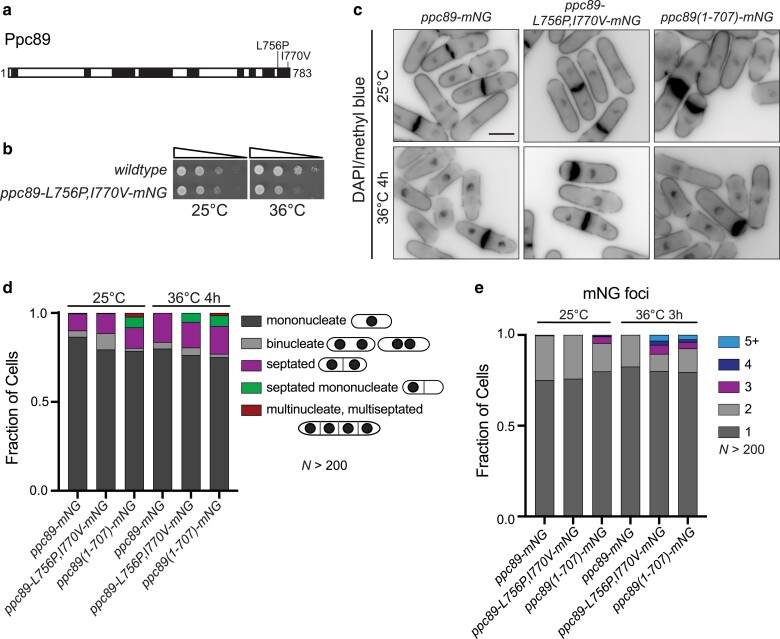
*Ppc89* alleles with disrupted α-helices 8 and 9 display SPB integrity and cell division defects. a) Schematic, drawn to scale, of Ppc89 and the AlphaFold predicted α-helices in black. The mutations encoded by the indicated allele are labeled. b) Ten-fold serial dilutions of the indicated strains grown at the indicated temperatures on YE agar for 2–3 days. c) Fixed-cell imaging of the indicated strains grown at 25°C and shifted to 36°C for 4 h. Samples were collected at both time points and stained with DAPI and MB. d) Quantification of the phenotypes from (c). *N* > 200 cells for each condition and strain from 3 independent experiments. e) Cells were grown at 25°C and were shifted to 36°C for 3 h and imaged at both time points. Quantification of Ppc89-mNG foci per cell in the indicated strains at the indicated temperatures. *N* > 200 cells for each condition and strain from 3 independent experiments. Scale bar, 5 µm.

*ppc89-4* contains several mutations outside of the Sid4 interaction region, some in the region truncated in *ppc89(1–707)*. To test whether the mutations mapping to α-helices 8 and 9 were responsible for the additional Sid4-independent phenotypes of *ppc89-4*, we constructed *ppc89-L756P,I770V* (Fig. 6a). *ppc89-L756P,I770V* was ts and accumulated cells with 1 nucleus and a septum (Fig. 6b–d), reminiscent of what we observed in *ppc89-4* cells. Moreover, we found that *ppc89(1–707)-mNG* cells accumulated mononucleated, septated cells and anucleate cell compartments (Fig. 6c and d) as well as multiple Ppc89 foci (Fig. 6e and Supplementary Fig. 4b).

**Fig. 7.**
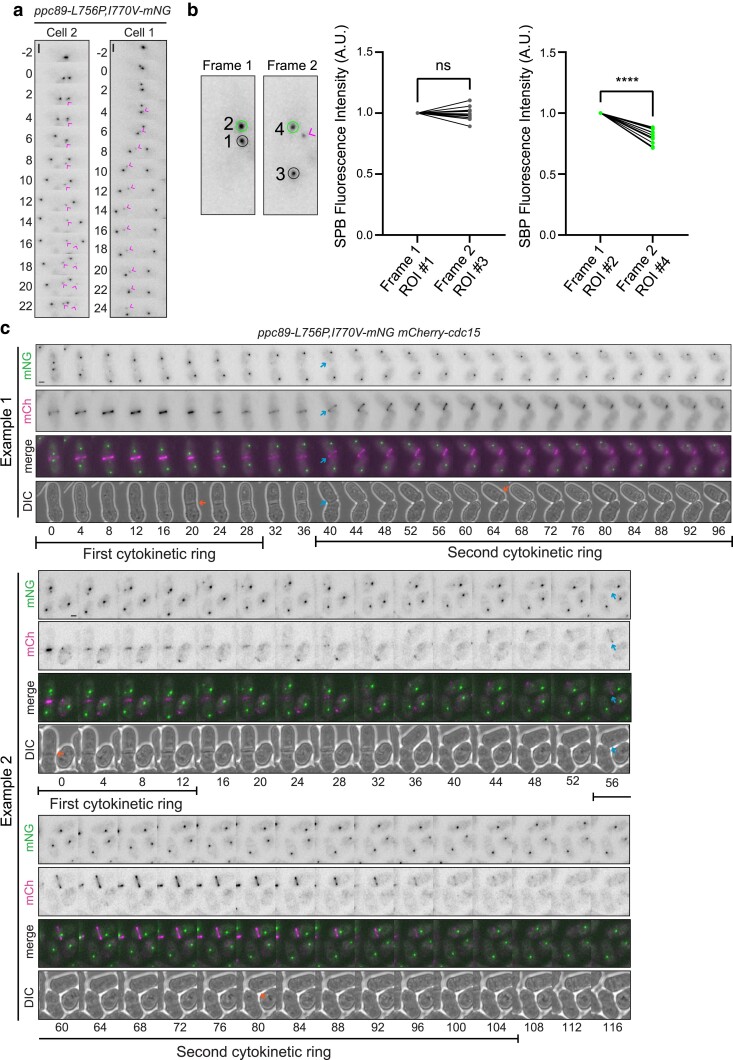
Disruption of Ppc89 α-helices 8 and 9 display SPB splitting and extra off-center septa. (a, c) Live-cell time-lapse microscopy of *ppc89-L756P,I770V-mNG mCherry-cdc15* cells. The cells were grown at 27°C and shifted to 36°C for 45 min prior to image acquisition and the cells were maintained at 36°C during imaging. Images were acquired every 2 min and representative montages of 5 movies containing 280 cells that entered and completed mitosis are shown. Every frame is shown in montages in (a) and every other frame is shown in the montages in (c). Time 0 in (a) is the initial frame of SPB separation and in (c), time 0 is the first frame of the movies. The magenta arrowheads in (a) indicate the extra Ppc89 foci. In (c), the blue arrows indicate where the second cytokinetic ring forms, and the orange arrows mark the formation of a septum. b) The fluorescence intensity of Ppc89-L756P,I770V-mNG at both SPBs was determined just prior to and immediately after Ppc89 foci appearance, schematized on the left. The left graph is the signal intensity of the SPB that did not appear to fragment. The graph on the right is the signal intensity of the SPB that gave rise to a focus. Each line represents the intensity change of a single SPB. *N* = 12 for each type of SPB from 3 independent experiments. Error bars represent mean ± SEM. Unfragmented SPBs from the graph with the gray data points, *P* = 0.76. ****P ≤ 0.0001; unpaired, 2-tailed Student's *t*-test. ns, no significance; ROI, region of interest. Scale bar, 2 µm.

To determine when the multiple Ppc89 foci and off-center septa developed relative to mitotic progression, we performed live-cell time-lapse imaging of cells expressing *ppc89-L756P,I770V-mNG* as an SPB marker and *mCherry-cdc15* as a cytokinetic ring marker. Interestingly we found that the additional foci of Ppc89-4, observed in 169/280 cells that progressed through mitosis during the movies, always formed during anaphase by splitting off from 1 of the 2 SPBs (Fig. 7a). By determining the intensity of the 2 SPBs before and after foci appeared, we confirmed that the fragments originated from 1 SPB because the fluorescence intensity of that SPB always diminished relative to the second SPB (Fig. 7b). We also determined that the rarer off-center septum phenotype (9 of 280 cells) arose because a second cytokinetic ring formed at the new cell end of 1 daughter cell after its birth (Fig. 7c). In these cells, the cytokinetic ring appeared not to fully disassemble and 1 daughter inherited this cortical material. Then, a second cytokinetic ring formed at variable times relative to the completion of the previous division where the first cytokinetic ring remnants were located (Fig. 7c). Taken together, these results implicate Ppc89 α-helices 8 and 9 in the regulation of SPB integrity and cytokinesis independent of its role in anchoring Sid4 and the SIN.

## Discussion

Ppc89 forms the core of the *S. pombe* SPB but how it connects to the many other essential SPB-localized proteins is not well understood. In this study, we developed conditional and truncation mutants of *ppc89* that allowed us to define both essential and nonessential elements of Ppc89. We refined our understanding of Ppc89's essential role in anchoring the SIN via the SIN scaffold Sid4, cemented our understanding of which SPB proteins require Ppc89–Sid4 interaction for SPB recruitment to the SPB, and implicated the very C-terminus of Ppc89 in ensuring proper coordination of cytokinesis and mitosis independently of SIN anchoring.

Ppc89 is one of the first proteins assembled into the daughter SPB, preceded only by the half-bridge protein Sfi1 and coincident with Sad1 (Bestul *et al.* 2017). While this timing could suggest that either Sfi1 and/or Sad1 recruits Ppc89, a direct linkage of Ppc89 with either protein has not been reported. Sid4 is recruited to the daughter SPB soon after Ppc89 (Bestul *et al.* 2017) and we have shown here that 2 predicted Ppc89 α-helices (6 and 7) directly bind Sid4. This allows the subsequent recruitment of centriolin-like Cdc11, and the remainder of the SIN signaling network assembles onto these 2 scaffolds. Interestingly, we found that the position of Sid4 at the SPB is at least somewhat flexible in that tethering Sid4 to the C-terminus of either Ppc89 or pericentrin Pcp1 results in functional Sid4-SIN assembly and function. The Pcp1 C-terminal PACT domain binds Ppc89 further toward its N-terminus including predicted α-helix 3 (Chen *et al.* 2024). Thus, it seems that the Pcp1 PACT domain and Sid4 may normally be in close proximity. Mto1 is also linked to Sid4–Cdc11 by an incompletely understood interaction mediated by the very C-terminal region of Mto1 (Samejima *et al.* 2010), providing another juxtaposition of regulators of cytokinesis and microtubule nucleation.

*ppc89-4* cells, but not *ppc89-3* cells, displayed 2 defects in addition to not anchoring Sid4 and the SIN. First, ∼17% of *ppc89-4* cells contained 3 or 5 Ppc89 foci, a phenotype also observed in a *ppc89* allele containing the subset of *ppc89-4* mutations within predicted α-helices 8 and 9 and in an allele truncated of these 2 α-helices. The additional Ppc89 foci break away from the SPB only during anaphase B resulting in a reduction of Ppc89 at the spindle pole. Second, at steady state, ∼6% of *ppc89-4* cells contained a single nucleus and an off-center septum. This phenotype was also shared with cells lacking Ppc89 α-helices 8 and 9 and duplicated in a *ppc89* mutant containing just the subset of the *ppc89-4* mutations within α-helices 8 and 9. We found this phenotype arose because 1 daughter cell underwent an additional round of septation soon after its birth. This was correlated with the incomplete disassembly of the cytokinetic ring. Cytokinetic ring material persisted at the cortex of 1 daughter cell into the next cell cycle and at variable times, another cytokinetic ring and septum formed at the position of this cortical material prior to the cell entering mitosis. We do not know if this phenotype is related to that of SPB fragmentation. To prevent additional rounds of septation uncoupled with mitosis, such as we observed in these *ppc89* mutants, SIN signaling is normally terminated at the end of anaphase (Johnson *et al.* 2012; Simanis 2015). However, the SIN is only active at 1 of the 2 SPBs during anaphase, and therefore only 1 daughter cell compartment needs to terminate SIN signaling after cytokinesis (Johnson *et al.* 2012; Simanis 2015). Termination of SIN signaling involves the loss of a SIN activator, Etd1, from the cortex of the daughter cell with active SIN at the SPB and requires that SIN signaling is asymmetric (Garcia-Cortes and McCollum 2009). Thus, although the function of Ppc89 α-helices 8 and 9 is not essential for viability at all temperatures and is not involved in anchoring the SIN itself, we hypothesize that 1 possible explanation for our observations is that this region of Ppc89 helps anchor a regulator that ensures proper asymmetric SIN signaling and its termination. The identity of such a factor remains to be determined in future experiments. Overall, however, our data indicate that Ppc89 not only tethers the SIN to the SPB but is also necessary for the integrity of the SPB and faithful coordination of cytokinesis with mitosis.

## Supplementary Material

jkae249_Supplementary_Data

## Data Availability

Strains and plasmids are available upon request. The data underlying Figs. 1–6 and Supplementary Figs. 1–4 are openly available in Mendeley Data at doi: 10.17632/ykzfrkz9v2.1, 10.17632/w3jctgv4fs.1, 10.17632/ymnk9wwvsw.1, 10.17632/9myyyp9878.1, 10.17632/cx4z2k63xj.1, 10.17632/gyxhnx6484.1, 10.17632/cgtn9t5ykb.1, and 10.17632/cxyhdbszg4.1. Supplemental material available at G3 online.

## References

[jkae249-B1] Abramson J, Adler J, Dunger J, Evans R, Green T, Pritzel A, Ronneberger O, Willmore L, Ballard AJ, Bambrick J, et al 2024. Accurate structure prediction of biomolecular interactions with AlphaFold 3. Nature. 630(8016):493–500. doi:10.1038/s41586-024-07487-w. 38718835 PMC11168924

[jkae249-B2] Alfa CE, Ducommun B, Beach D, Hyams JS. 1990. Distinct nuclear and spindle pole body population of cyclin-cdc2 in fission yeast. Nature. 347(6294):680–682. doi:10.1038/347680a0. 1699136

[jkae249-B3] Bahler J, Steever AB, Wheatley S, Wang Y, Pringle JR, Gould KL, McCollum D. 1998. Role of polo kinase and Mid1p in determining the site of cell division in fission yeast. J Cell Biol. 143(6):1603–1616. doi:10.1083/jcb.143.6.1603. 9852154 PMC2132972

[jkae249-B4] Bahler J, Wu JQ, Longtine MS, Shah NG, McKenzie A 3rd, Steever AB, Wach A, Philippsen P, Pringle JR. 1998. Heterologous modules for efficient and versatile PCR-based gene targeting in *Schizosaccharomyces pombe*. Yeast. 14(10):943–951.doi:10.1002/(SICI)1097-0061(199807)14:10<943::AID-YEA292>3.0.CO;2-Y. 9717240

[jkae249-B5] Balasubramanian MK, McCollum D, Chang L, Wong KC, Naqvi NI, He X, Sazer S, Gould KL. 1998. Isolation and characterization of new fission yeast cytokinesis mutants. Genetics. 149(3):1265–1275. doi:10.1093/genetics/149.3.1265. 9649519 PMC1460233

[jkae249-B6] Bestul AJ, Yu Z, Unruh JR, Jaspersen SL. 2017. Molecular model of fission yeast centrosome assembly determined by superresolution imaging. J Cell Biol. 216(8):2409–2424. doi:10.1083/jcb.201701041. 28619713 PMC5551712

[jkae249-B7] Bestul AJ, Yu Z, Unruh JR, Jaspersen SL. 2021. Redistribution of centrosomal proteins by centromeres and Polo kinase controls partial nuclear envelope breakdown in fission yeast. Mol Biol Cell. 32(16):1487–1500. doi:10.1091/mbc.E21-05-0239. 34133218 PMC8351742

[jkae249-B8] Bornens M . 2021. Centrosome organization and functions. Curr Opin Struct Biol. 66:199–206. doi:10.1016/j.sbi.2020.11.002. 33338884

[jkae249-B9] Bouhlel IB, Ohta M, Mayeux A, Bordes N, Dingli F, Boulanger J, Casquillas GV, Loew D, Tran PT, Sato M, et al 2015. Cell cycle control of spindle pole body duplication and splitting by sfi1 and Cdc31 in fission yeast. J Cell Sci. 128(8):1481–1493. doi:10.1242/jcs.159657. 25736294

[jkae249-B10] Bullitt E, Rout MP, Kilmartin JV, Akey CW. 1997. The yeast spindle pole body is assembled around a central crystal of Spc42p. Cell. 89(7):1077–1086. doi:10.1016/S0092-8674(00)80295-0. 9215630

[jkae249-B11] Cavanaugh AM, Jaspersen SL. 2017. Big lessons from little yeast: budding and fission yeast centrosome structure, duplication, and function. Annu Rev Genet. 51(1):361–383. doi:10.1146/annurev-genet-120116-024733. 28934593

[jkae249-B12] Chang L, Gould KL. 2000. Sid4p is required to localize components of the septation initiation pathway to the spindle pole body in fission yeast. Proc Natl Acad Sci U S A. 97(10):5249–5254. doi:10.1073/pnas.97.10.5249. 10805785 PMC25814

[jkae249-B13] Chavali PL, Putz M, Gergely F. 2014. Small organelle, big responsibility: the role of centrosomes in development and disease. Philos Trans R Soc Lond B Biol Sci. 369(1650):20130468. doi:10.1098/rstb.2013.0468. 25047622 PMC4113112

[jkae249-B14] Chen JS, Igarashi MG, Ren L, Hanna SM, Turner LA, McDonald NA, Beckley JR, Willet AH, Gould KL. 2024. The core spindle pole body scaffold Ppc89 links the pericentrin orthologue Pcp1 to the fission yeast spindle pole body via an evolutionarily conserved interface. Mol Biol Cell. 35(8):ar112. doi:10.1091/mbc.E24-05-0220. 38985524 PMC11321043

[jkae249-B15] Cueille N, Salimova E, Esteban V, Blanco M, Moreno S, Bueno A, Simanis V. 2001. Flp1, a fission yeast orthologue of the *S. cerevisiae* CDC14 gene, is not required for cyclin degradation or rum1p stabilisation at the end of mitosis. J Cell Sci. 114(14):2649–2664. doi:10.1242/jcs.114.14.2649. 11683392

[jkae249-B16] Cullati SN, Gould KL. 2019. Spatiotemporal regulation of the Dma1-mediated mitotic checkpoint coordinates mitosis with cytokinesis. Curr Genet. 65(3):663–668. doi:10.1007/s00294-018-0921-x. 30600396 PMC6511297

[jkae249-B17] Decottignies A, Zarzov P, Nurse P. 2001. In vivo localisation of fission yeast cyclin-dependent kinase cdc2p and cyclin B cdc13p during mitosis and meiosis. J Cell Sci. 114(14):2627–2640. doi:10.1242/jcs.114.14.2627. 11683390

[jkae249-B18] Ding R, West RR, Morphew DM, Oakley BR, McIntosh JR. 1997. The spindle pole body of *Schizosaccharomyces pombe* enters and leaves the nuclear envelope as the cell cycle proceeds. Mol Biol Cell. 8(8):1461–1479. doi:10.1091/mbc.8.8.1461. 9285819 PMC276170

[jkae249-B19] Flory MR, Morphew M, Joseph JD, Means AR, Davis TN. 2002. Pcp1p, an Spc110p-related calmodulin target at the centrosome of the fission yeast *Schizosaccharomyces pombe*. Cell Growth Differ. 13:47–58. 11864908

[jkae249-B20] Fong CS, Sato M, Toda T. 2010. Fission yeast Pcp1 links polo kinase-mediated mitotic entry to gamma-tubulin-dependent spindle formation. EMBO J. 29(1):120–130. doi:10.1038/emboj.2009.331. 19942852 PMC2788132

[jkae249-B21] Forsburg SL, Rhind N. 2006. Basic methods for fission yeast. Yeast. 23(3):173–183. doi:10.1002/yea.1347. 16498704 PMC5074380

[jkae249-B22] Fu J, Hagan IM, Glover DM. 2015. The centrosome and its duplication cycle. Cold Spring Harb Perspect Biol. 7(2):a015800. doi:10.1101/cshperspect.a015800. 25646378 PMC4315929

[jkae249-B23] Ganem NJ, Storchova Z, Pellman D. 2007. Tetraploidy, aneuploidy and cancer. Curr Opin Genet Dev. 17(2):157–162. doi:10.1016/j.gde.2007.02.011. 17324569

[jkae249-B24] Garcia-Cortes JC, McCollum D. 2009. Proper timing of cytokinesis is regulated by *Schizosaccharomyces pombe* Etd1. J Cell Biol. 186(5):739–753. doi:10.1083/jcb.200902116. 19736319 PMC2742193

[jkae249-B25] Godinho SA, Picone R, Burute M, Dagher R, Su Y, Leung CT, Polyak K, Brugge JS, Théry M, Pellman D. 2014. Oncogene-like induction of cellular invasion from centrosome amplification. Nature. 510(7503):167–171. doi:10.1038/nature13277. 24739973 PMC4061398

[jkae249-B26] Gould KL, Moreno S, Owen DJ, Sazer S, Nurse P. 1991. Phosphorylation at Thr167 is required for *Schizosaccharomyces pombe* p34cdc2 function. Embo J. 10(11):3297–3309. doi:10.1002/j.1460-2075.1991.tb04894.x. 1655416 PMC453056

[jkae249-B27] Guertin DA, Venkatram S, Gould KL, McCollum D. 2002. Dma1 prevents mitotic exit and cytokinesis by inhibiting the septation initiation network (SIN). Dev Cell. 3(6):779–790. doi:10.1016/S1534-5807(02)00367-2. 12479804

[jkae249-B28] Hagan I, Yanagida M. 1995. The product of the spindle formation gene sad1+ associates with the fission yeast spindle pole body and is essential for viability. J Cell Biol. 129(4):1033–1047. doi:10.1083/jcb.129.4.1033. 7744953 PMC2120497

[jkae249-B29] Ito D, Bettencourt-Dias M. 2018. Centrosome remodelling in evolution. Cells. 7(7):71. doi:10.3390/cells7070071. 29986477 PMC6070874

[jkae249-B30] James P, Halladay J, Craig EA. 1996. Genomic libraries and a host strain designed for highly efficient two-hybrid selection in yeast. Genetics. 144(4):1425–1436. doi:10.1093/genetics/144.4.1425. 8978031 PMC1207695

[jkae249-B31] Janson ME, Setty TG, Paoletti A, Tran PT. 2005. Efficient formation of bipolar microtubule bundles requires microtubule-bound gamma-tubulin complexes. J Cell Biol. 169(2):297–308. doi:10.1083/jcb.200410119. 15837798 PMC2171869

[jkae249-B32] Jaspersen SL . 2021. Anatomy of the fungal microtubule organizing center, the spindle pole body. Curr Opin Struct Biol. 66:22–31. doi:10.1016/j.sbi.2020.09.008. 33113389 PMC7965227

[jkae249-B33] Johnson AE, Chen JS, Gould KL. 2013. CK1 is required for a mitotic checkpoint that delays cytokinesis. Curr Biol. 23(19):1920–1926. doi:10.1016/j.cub.2013.07.077. 24055157 PMC4078987

[jkae249-B34] Johnson AE, McCollum D, Gould KL. 2012. Polar opposites: fine-tuning cytokinesis through SIN asymmetry. Cytoskeleton. 69(10):686–699. doi:10.1002/cm.21044. 22786806 PMC3478943

[jkae249-B35] Jumper J, Evans R, Pritzel A, Green T, Figurnov M, Ronneberger O, Tunyasuvunakool K, Bates R, Žídek A, Potapenko A, et al 2021. Highly accurate protein structure prediction with AlphaFold. Nature. 596(7873):583–589. doi:10.1038/s41586-021-03819-2. 34265844 PMC8371605

[jkae249-B36] Keeney JB, Boeke JD. 1994. Efficient targeted integration at leu1-32 and ura4-294 in *Schizosaccharomyces pombe*. Genetics. 136(3):849–856. doi:10.1093/genetics/136.3.849. 8005439 PMC1205890

[jkae249-B37] Kilmartin JV . 2003. Sfi1p has conserved centrin-binding sites and an essential function in budding yeast spindle pole body duplication. J Cell Biol. 162(7):1211–1221. doi:10.1083/jcb.200307064. 14504268 PMC2173958

[jkae249-B38] Kilmartin JV . 2014. Lessons from yeast: the spindle pole body and the centrosome. Philos Trans R Soc Lond B Biol Sci. 369(1650):20130456. doi:10.1098/rstb.2013.0456. 25047610 PMC4113100

[jkae249-B39] Krapp A, Schmidt S, Cano E, Simanis V. 2001. *S. pombe* cdc11p, together with sid4p, provides an anchor for septation initiation network proteins on the spindle pole body. Curr Biol. 11(20):1559–1568. doi:10.1016/S0960-9822(01)00478-X. 11676915

[jkae249-B40] Langlois-Lemay L, D'Amours D. 2022. Moonlighting at the poles: non-canonical functions of centrosomes. Front Cell Dev Biol. 10:930355. doi:10.3389/fcell.2022.930355. 35912107 PMC9329689

[jkae249-B41] Lee IJ, Wang N, Hu W, Schott K, Bähler J, Giddings TH Jr, Pringle JR, Du L-L, Wu J-Q. 2014. Regulation of spindle pole body assembly and cytokinesis by the centrin-binding protein Sfi1 in fission yeast. Mol Biol Cell. 25(18):2735–2749. doi:10.1091/mbc.e13-11-0699. 25031431 PMC4161509

[jkae249-B42] Li S, Sandercock AM, Conduit P, Robinson CV, Williams RL, Kilmartin JV. 2006. Structural role of Sfi1p-centrin filaments in budding yeast spindle pole body duplication. J Cell Biol. 173(6):867–877. doi:10.1083/jcb.200603153. 16785321 PMC2063913

[jkae249-B43] Lin M, Xie SS, Chan KY. 2022. An updated view on the centrosome as a cell cycle regulator. Cell Div. 17(1):1. doi:10.1186/s13008-022-00077-0. 35164835 PMC8842576

[jkae249-B44] Lin TC, Neuner A, Schiebel E. 2015. Targeting of gamma-tubulin complexes to microtubule organizing centers: conservation and divergence. Trends Cell Biol. 25(5):296–307. doi:10.1016/j.tcb.2014.12.002. 25544667

[jkae249-B45] Lynch EM, Groocock LM, Borek WE, Sawin KE. 2014. Activation of the gamma-tubulin complex by the mto1/2 complex. Curr Biol. 24(8):896–903. doi:10.1016/j.cub.2014.03.006. 24704079 PMC3989768

[jkae249-B46] Mirdita M, Schutze K, Moriwaki Y, Heo L, Ovchinnikov S, Steinegger M. 2022. ColabFold: making protein folding accessible to all. Nat Methods. 19(6):679–682. doi:10.1038/s41592-022-01488-1. 35637307 PMC9184281

[jkae249-B47] Moreno S, Klar A, Nurse P. 1991. Molecular genetic analysis of fission yeast *Schizosaccharomyces pombe*. Methods Enzymol. 194:795–823. doi:10.1016/0076-6879(91)94059-L. 2005825

[jkae249-B48] Morrell JL, Nichols CB, Gould KL. 2004. The GIN4 family kinase, Cdr2p, acts independently of septins in fission yeast. J Cell Sci. 117(22):5293–5302. doi:10.1242/jcs.01409. 15454577

[jkae249-B49] Morrell JL, Tomlin GC, Rajagopalan S, Venkatram S, Feoktistova AS, Tasto JJ, Mehta S, Jennings JL, Link A, Balasubramanian MK, et al 2004. Sid4p-Cdc11p assembles the septation initiation network and its regulators at the *S. pombe* SPB. Curr Biol. 14(7):579–584. doi:10.1016/j.cub.2004.03.036. 15062098

[jkae249-B50] Mulvihill DP, Petersen J, Ohkura H, Glover DM, Hagan IM. 1999. Plo1 kinase recruitment to the spindle pole body and its role in cell division in *Schizosaccharomyces pombe*. Mol Biol Cell. 10(8):2771–2785. doi:10.1091/mbc.10.8.2771. 10436027 PMC25513

[jkae249-B51] Paoletti A, Bordes N, Haddad R, Schwartz CL, Chang F, Bornens M. 2003. Fission yeast cdc31p is a component of the half-bridge and controls SPB duplication. Mol Biol Cell. 14(7):2793–2808. doi:10.1091/mbc.e02-10-0661. 12857865 PMC165677

[jkae249-B52] Roberts-Galbraith RH, Chen JS, Wang J, Gould KL. 2009. The SH3 domains of two PCH family members cooperate in assembly of the *Schizosaccharomyces pombe* contractile ring. J Cell Biol. 184(1):113–127. doi:10.1083/jcb.200806044. 19139265 PMC2615086

[jkae249-B53] Rosenberg JA, Tomlin GC, McDonald WH, Snydsman BE, Muller EG, Yates JR 3rd, Gould KL. 2006. Ppc89 links multiple proteins, including the septation initiation network, to the core of the fission yeast spindle-pole body. Mol Biol Cell. 17(9):3793–3805. doi:10.1091/mbc.e06-01-0039. 16775007 PMC1593159

[jkae249-B54] Rothbauer U, Zolghadr K, Muyldermans S, Schepers A, Cardoso MC, Leonhardt H. 2008. A versatile nanotrap for biochemical and functional studies with fluorescent fusion proteins. Mol Cell Proteomics. 7(2):282–289. doi:10.1074/mcp.M700342-MCP200. 17951627

[jkae249-B55] Rothbauer U, Zolghadr K, Tillib S, Nowak D, Schermelleh L, Gahl A, Backmann N, Conrath K, Muyldermans S, Cardoso MC, et al 2006. Targeting and tracing antigens in live cells with fluorescent nanobodies. Nat Methods. 3(11):887–889. doi:10.1038/nmeth953. 17060912

[jkae249-B56] Ruthnick D, Schiebel E. 2016. Duplication of the yeast spindle pole body once per cell cycle. Mol Cell Biol. 36(9):1324–1331. doi:10.1128/MCB.00048-16. 26951196 PMC4836218

[jkae249-B57] Ruthnick D, Vitale J, Neuner A, Schiebel E. 2021. The N-terminus of Sfi1 and yeast centrin Cdc31 provide the assembly site for a new spindle pole body. J Cell Biol. 220(3):e202004196. doi:10.1083/jcb.202004196. 33523111 PMC7852455

[jkae249-B58] Ryniawec JM, Rogers GC. 2021. Centrosome instability: when good centrosomes go bad. Cell Mol Life Sci. 78(21–22):6775–6795. doi:10.1007/s00018-021-03928-1. 34476544 PMC8560572

[jkae249-B59] Salimova E, Sohrmann M, Fournier N, Simanis V. 2000. The *S. pombe* orthologue of the *S. cerevisiae mob1* gene is essential and functions in signalling the onset of septum formation. J Cell Sci. 113(10):1695–1704. doi:10.1242/jcs.113.10.1695. 10769201

[jkae249-B60] Samejima I, Lourenco PC, Snaith HA, Sawin KE. 2005. Fission yeast mto2p regulates microtubule nucleation by the centrosomin-related protein mto1p. Mol Biol Cell. 16(6):3040–3051. doi:10.1091/mbc.e04-11-1003. 15659644 PMC1142446

[jkae249-B61] Samejima I, Miller VJ, Rincon SA, Sawin KE. 2010. Fission yeast Mto1 regulates diversity of cytoplasmic microtubule organizing centers. Curr Biol. 20(21):1959–1965. doi:10.1016/j.cub.2010.10.006. 20970338 PMC2989437

[jkae249-B62] Sawin KE, Lourenco PC, Snaith HA. 2004. Microtubule nucleation at non-spindle pole body microtubule-organizing centers requires fission yeast centrosomin-related protein mod20p. Curr Biol. 14(9):763–775. doi:10.1016/j.cub.2004.03.042. 15120067

[jkae249-B63] Schindelin J, Arganda-Carreras I, Frise E, Kaynig V, Longair M, Pietzsch T, Preibisch S, Rueden C, Saalfeld S, Schmid B, et al 2012. Fiji: an open-source platform for biological-image analysis. Nat Methods. 9(7):676–682. doi:10.1038/nmeth.2019. 22743772 PMC3855844

[jkae249-B64] Simanis V . 2015. Pombe's thirteen - control of fission yeast cell division by the septation initiation network. J Cell Sci. 128(8):1465–1474. doi:10.1242/jcs.094821. 25690009

[jkae249-B65] Singh NS, Shao N, McLean JR, Sevugan M, Ren L, Chew TG, Bimbo A, Sharma R, Tang X, Gould KL, et al 2011. SIN-inhibitory phosphatase complex promotes Cdc11p dephosphorylation and propagates SIN asymmetry in fission yeast. Curr Biol. 21(23):1968–1978. doi:10.1016/j.cub.2011.10.051. 22119525 PMC4167312

[jkae249-B66] Tang NH, Fong CS, Masuda H, Jourdain I, Yukawa M, Toda T. 2019. Generation of temperature sensitive mutations with error-prone PCR in a gene encoding a component of the spindle pole body in fission yeast. Biosci Biotechnol Biochem. 83(9):1717–1720. doi:10.1080/09168451.2019.1611414. 31042107

[jkae249-B67] Tomlin GC, Morrell JL, Gould KL. 2002. The spindle pole body protein Cdc11p links Sid4p to the fission yeast septation initiation network. Mol Biol Cell. 13(4):1203–1214. doi:10.1091/mbc.01-09-0455. 11950932 PMC102262

[jkae249-B68] Trautmann S, Wolfe BA, Jorgensen P, Tyers M, Gould KL, McCollum D. et al 2001. Fission yeast Clp1p phosphatase regulates G2/M transition and coordination of cytokinesis with cell cycle progression. Curr Biol. 11(12):931–940. doi:10.1016/S0960-9822(01)00268-8. 11448769

[jkae249-B69] Uzawa S, Li F, Jin Y, McDonald KL, Braunfeld MB, Agard DA, Cande WZ. 2004. Spindle pole body duplication in fission yeast occurs at the G1/S boundary but maturation is blocked until exit from S by an event downstream of cdc10+. Mol Biol Cell. 15(12):5219–5230. doi:10.1091/mbc.e04-03-0255. 15385623 PMC532005

[jkae249-B70] Varadi M, Anyango S, Deshpande M, Nair S, Natassia C, Yordanova G, Yuan D, Stroe O, Wood G, Laydon A, et al 2022. AlphaFold protein structure database: massively expanding the structural coverage of protein-sequence space with high-accuracy models. Nucleic Acids Res. 50(D1):D439–D444. doi:10.1093/nar/gkab1061. 34791371 PMC8728224

[jkae249-B71] Vasquez-Limeta A, Loncarek J. 2021. Human centrosome organization and function in interphase and mitosis. Semin Cell Dev Biol. 117:30–41. doi:10.1016/j.semcdb.2021.03.020. 33836946 PMC8465925

[jkae249-B72] Venkatram S, Jennings JL, Link A, Gould KL. 2005. Mto2p, a novel fission yeast protein required for cytoplasmic microtubule organization and anchoring of the cytokinetic actin ring. Mol Biol Cell. 16(6):3052–3063. doi:10.1091/mbc.e04-12-1043. 15800064 PMC1142447

[jkae249-B73] Venkatram S, Tasto JJ, Feoktistova A, Jennings JL, Link AJ, Gould KL. 2004. Identification and characterization of two novel proteins affecting fission yeast gamma-tubulin complex function. Mol Biol Cell. 15(5):2287–2301. doi:10.1091/mbc.e03-10-0728. 15004232 PMC404023

[jkae249-B74] Viswanath S, Bonomi M, Kim SJ, Klenchin VA, Taylor KC, Yabut KC, Umbreit NT, Van Epps HA, Meehl J, Jones MH, et al 2017. The molecular architecture of the yeast spindle pole body core determined by Bayesian integrative modeling. Mol Biol Cell. 28(23):3298–3314. doi:10.1091/mbc.e17-06-0397. 28814505 PMC5687031

[jkae249-B75] Waters JC . 2009. Accuracy and precision in quantitative fluorescence microscopy. J Cell Biol. 185(7):1135–1148. doi:10.1083/jcb.200903097. 19564400 PMC2712964

