# Supplementary material for: New mutations in the core Schizosaccharomyces pombe spindle pole body scaffold Ppc89 reveal separable functions in regulating cell division
Source: G3 (Bethesda). 2024 Oct 29;15(1):jkae249. doi: 10.1093/g3journal/jkae249 (PMC11708228; doi:10.1093/g3journal/jkae249)
Supplement: jkae249_Supplementary_Data [file jkae249_supplementary_data.pdf]

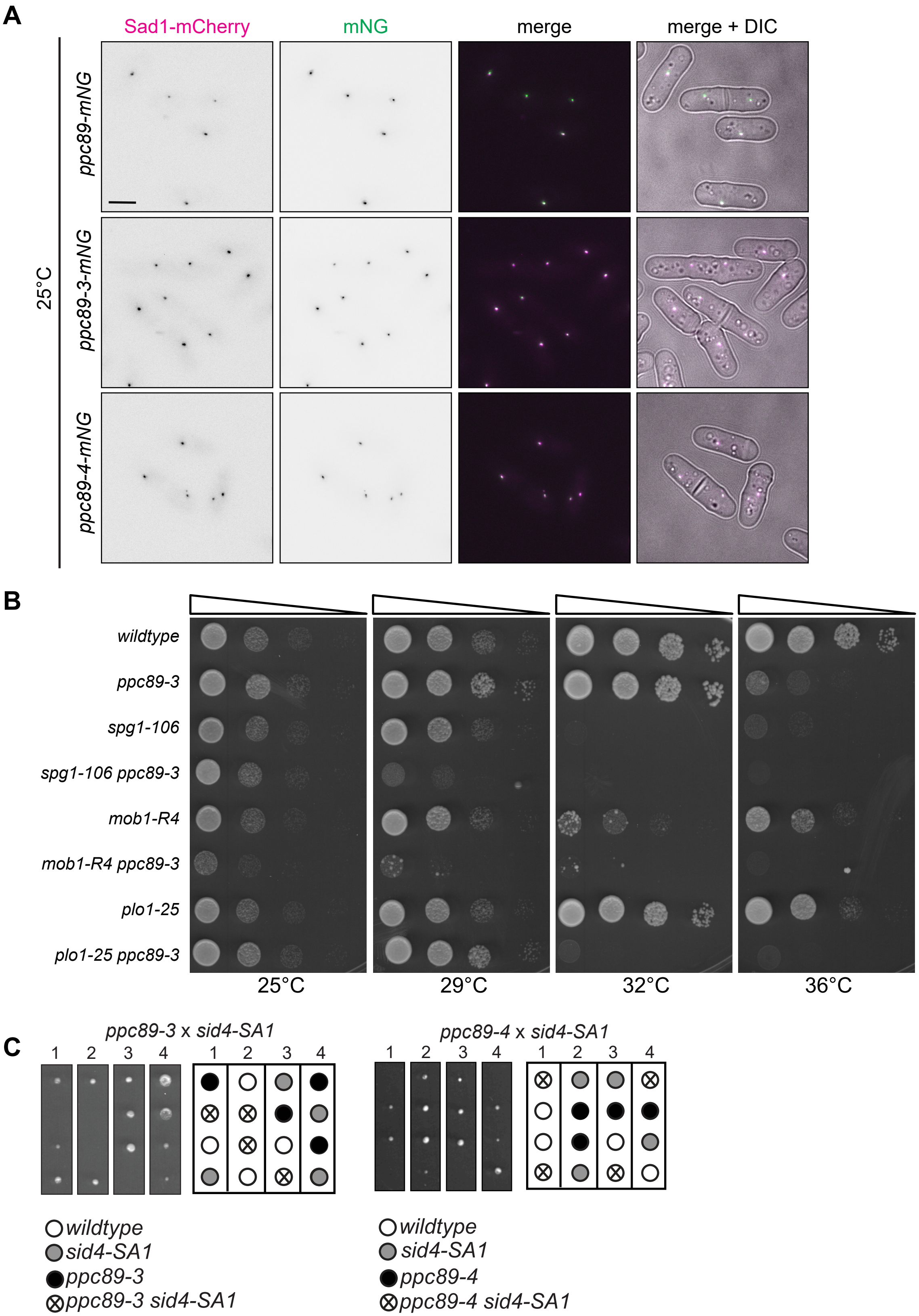
A
Sad1-mCherry
mNG
merge
merge + DIC
25°C
ppc89-mNG
ppc89-3-mNG
ppc89-4-mNG
B
wildtype
ppc89-3
spg1-106
spg1-106 ppc89-3
mob1-R4
mob1-R4 ppc89-3
plo1-25
plo1-25 ppc89-3
25°C
29°C
32°C
36°C
C
ppc89-3 x sid4-SA1
1 2 3 4 1 2 3 4
wildtype
sid4-SA1
ppc89-3
ppc89-3 sid4-SA1
ppc89-4 x sid4-SA1
1 2 3 4 1 2 3 4
wildtype
sid4-SA1
ppc89-4
ppc89-4 sid4-SA1

Figure S1

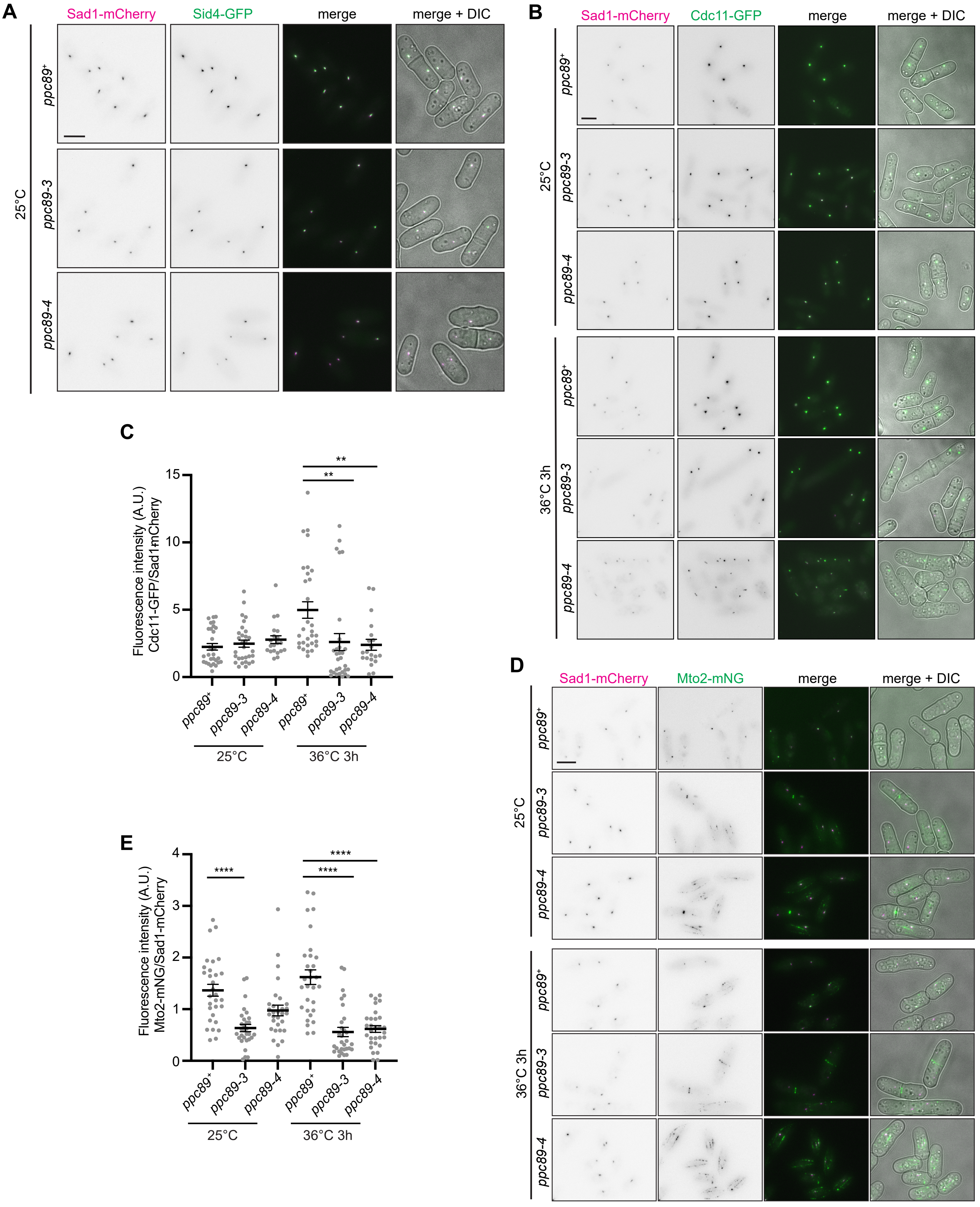
A
Sad1-mCherry
Sid4-GFP
merge
merge + DIC
25°C
ppc89+
ppc89-3
ppc89-4
B
Sad1-mCherry
Cdc11-GFP
merge
merge + DIC
25°C
36°C 3h
C
Fluorescence intensity (A.U.)
Cdc11-GFP/Sad1-mCherry
**
**
15
10
5
0
ppc89+
ppc89-3
ppc89-4
25°C
36°C 3h
D
Sad1-mCherry
Mto2-mNG
merge
merge + DIC
25°C
36°C 3h
E
Fluorescence intensity (A.U.)
Mto2-mNG/Sad1-mCherry
****
4
3
2
1
0

Figure S2

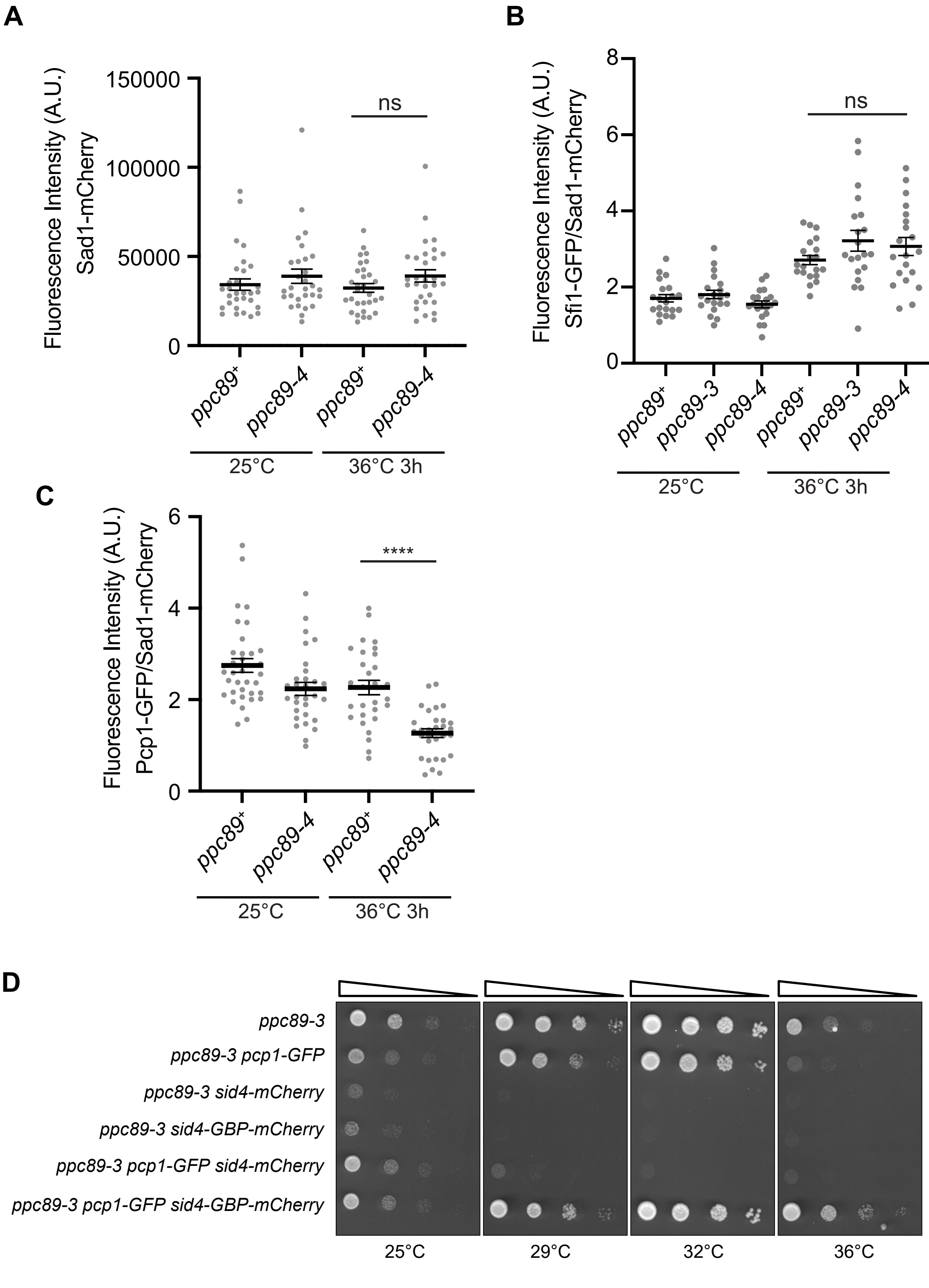

A
Fluorescence Intensity (A.U.) Sad1-mCherry
150000
100000
50000
0
ns
ppc89+
ppc89-4
ppc89+
ppc89-4
25°C
36°C 3h
B
Fluorescence Intensity (A.U.) Sfi1-GFP/Sad1-mCherry
8
6
4
2
0
ns
ppc89+
ppc89-3
ppc89-4
ppc89+
ppc89-3
ppc89-4
25°C
36°C 3h
C
Fluorescence Intensity (A.U.) Pcp1-GFP/Sad1-mCherry
6
4
2
0
****
ppc89+
ppc89-4
ppc89+
ppc89-4
25°C
36°C 3h
D
ppc89-3
ppc89-3 pcp1-GFP
ppc89-3 sid4-mCherry
ppc89-3 sid4-GBP-mCherry
ppc89-3 pcp1-GFP sid4-mCherry
ppc89-3 pcp1-GFP sid4-GBP-mCherry
25°C
29°C
32°C
36°C

Figure S3

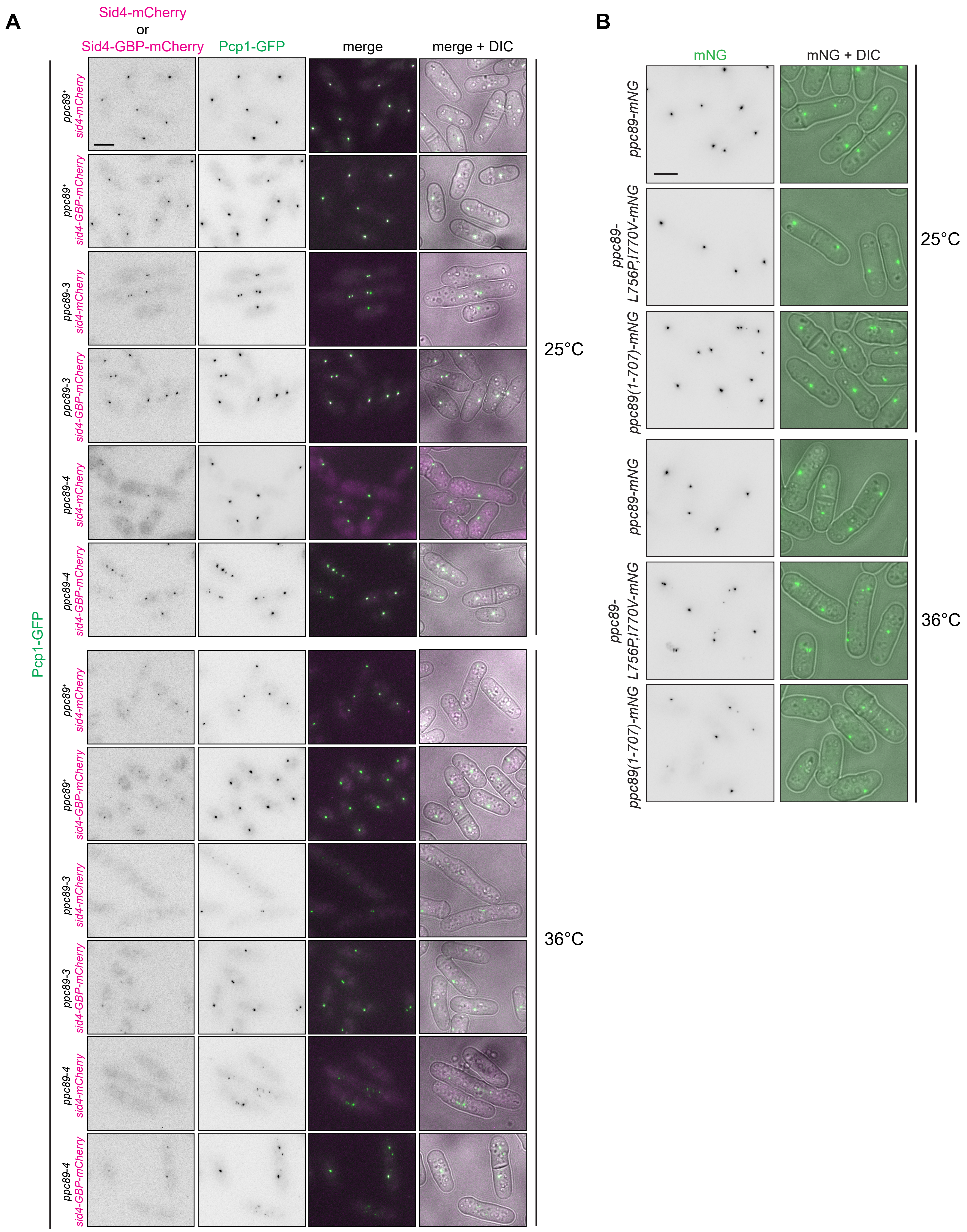
A
Sid4-mCherry
or
Sid4-GBP-mCherry
Pcp1-GFP
merge
merge + DIC
Pcp1-GFP
ppc89+ sid4-mCherry
ppc89+ sid4-GBP-mCherry
ppc89-3 sid4-mCherry
ppc89-3 sid4-GBP-mCherry
ppc89-4 sid4-mCherry
ppc89-4 sid4-GBP-mCherry
25°C
36°C
B
mNG
mNG + DIC
ppc89-mNG
ppc89-L756P,I770V-mNG
ppc89(1-707)-mNG
25°C
36°C

Figure S4

Supplemental Figure Legends

Figure S1. Genetic analysis and *ppc89* fragment rescue of *ppc89 ts* alleles. (A) Live-cell imaging of cells expressing Ppc89-mNG, Ppc89-3-mNG and Ppc89-4-mNG with Sad1-mCherry. Cells were grown at 25°C. Scale Bar, 5 µm. **(B)** Ten-fold serial dilutions of the indicated strains grown at the indicated temperatures on YE agar for 2-3 days. **(C)** Representative tetrads from the indicated genetic crosses.

Figure S2. Localization of SIN components in *ppc89-3.* (A) Live-cell imaging of cells expressing Sid4-GFP Sad1-mCherry in $ppc89^{+}$ or *ppc89-3*, or *ppc89-4* background. Cells were grown at 25°C. **(B)** Live-cell imaging of cells expressing Cdc11-GFP Sad1-mCherry in $ppc89^{+}$ or *ppc89-3*, or *ppc89-4* background. Cells were grown at 25°C and were shifted to 36°C for 3 hours. Images were acquired at both time points. **(C)** Quantification of SPB fluorescence intensity of strains from B. Cdc11-GFP SPB fluorescence intensity was divided by Sad1-mCherry SPB fluorescence intensity. $N \geq 20$ for each condition and strain from two independent experiments. Error bars represent mean ± SEM. *P*-values were determined with one-way ANOVA with Tukey's post hoc test, $^{**}P < 0.01$. **(D)** Live-cell imaging of cells expressing Mto2-mNG Sad1-mCherry in $ppc89^{+}$, *ppc89-3* or *ppc89-4* background. Cells were grown at 25°C and were shifted to 36°C for 3 hours. Images were acquired at both time points. **(E)** Quantification of SBP fluorescence intensity of mitotic cells from D. Mto2-mNG SPB fluorescence intensity was divided by Sad1-mCherry SPB fluorescence intensity. $N = 30$ for each condition and strain from three independent experiments. Error bars represent mean ± SEM. *P*-

values were determined with one-way ANOVA with Tukey's post hoc test, $****P \leq 0.0001$. Scale Bars, 5 µm.

Figure S3. Localization of Sad1 and Pcp1 in *ppc89-4* and Sid4 tethered to Pcp1 rescues *ppc89-3*. (A) Quantification of Sad1-mCherry SPB fluorescence intensity from Figs 1E and S1B. Error bars represent mean ± SEM. One-way ANOVA with Tukey's post hoc test, *ppc89*$^+$ vs. *ppc89-4* at 36°C, $P = 0.47$. $N = 30$ for each condition and strain from three independent experiments. **(B)** Quantification of Sfi1-GFP SPB fluorescence intensity divided by Sad1-mCherry SPB fluorescence intensity. Error bars represent mean ± SEM. p values were determined with one-way ANOVA with Tukey's post hoc test. *ppc89*$^+$ vs. *ppc89-3* at 36°C, $P = 0.30$. *ppc89*$^+$ vs. *ppc89-4* at 36°C, $P = 0.68$. $N = 20$ for each from two independent experiments. **(C)** Quantification of Pcp1-GFP SPB fluorescence intensity divided by Sad1-mCherry SPB fluorescence intensity. Error bars represent mean ± SEM. p values were determined with a one-way ANOVA with Tukey's post hoc test, $****P \leq 0.0001$. $N = 20$ for each condition and strain from two independent experiments. ns = no significance. **(D)** Ten-fold serial dilutions of the indicated strains grown at the indicated temperatures on YE agar for 2-3 days.

Figure S4. Imaging of Sid4 tethered stains in *ppc89-3* and *ppc89-4*. (A-B) Live-cell imaging of the indicated strains. Cells were grown at 25°C and were shifted to 36°C for 3 hours. Images were acquired at both time points. Scale Bar, 5 µm.

TABLE S1 *S. pombe* strains used in this study

Strain	Genotype	Source
**Figure 1**		
KGY246	*ade6-M210 ura4-D18 leu1-32 h*$^{-}$	Lab stock
KGY5578-2	*ppc89-2:kanMX6 ade6-M210 leu1-32 ura4-D18 h*$^{+}$	This study
KGY5590-2	*ppc89-3:kanMX6 ade6-M210 ura4-D18 leu1-32 h*$^{-}$	This study
KGY5580-2	*ppc89-4:kanMX6 ade6-M210 leu1-32 ura4-D18 h*$^{+}$	This study
KGY6493-2	*ppc89-mNG:hphMX6 sad1-mCherry:kanMX6 ade6-M21X ura4-D18 leu1-32 h*$^{+}$	This study
KGY6502-2	*ppc89-3-mNG:hphMX6 sad1-mCherry:kanMX6 ade6-M21X ura4-D18 leu1-32 h*$^{+}$	This study
KGY8133-2	*ppc89-4-mNG:hphMX6 sad1-mCherry:kanMX6 ade6-M21X ura4-D18 leu1-32 h*$^{-}$	This study
**Figure 2**		
KGY8013-2	*sad1-mCherry:natMX6 sid4-GFP:kanMX6 ade6-M21X ura4-D18 leu1-32 h*$^{-}$	This study
KGY8010-2	*ppc89-3:kanMX6 sad1-mCherry:natMX6 sid4-GFP:kanMX6 ade6-M21X ura4-D18 leu1-32 h*$^{-}$	This study
KGY8041-2	*ppc89-4:kanMX6 sad1-mCherry:natMX6 sid4-GFP:kanMX6 ade6-M21X ura4-D18 leu1-32 h*$^{-}$	This study
**Figure 3**		
KGY4291	*ppc89-*Myc_{13}*:kanMX6 ade6-M210 ura4-D18 leu1-32 h*$^{-}$	Lab Stock
KGY9421-2	*ppc89(1-707)-*Myc_{13}*:kanMX6 ade6-M210 ura4-D18 leu1-32 h*$^{-}$	This study
KGY6344-2	*ppc89-mNG:hphMX6 ade6-M21X ura4-D18 leu1-32 h*$^{-}$	This study
KGY8644-2	*ppc89(1-707)-mNG:hphMX6 ade6-M21X ura4-D18 leu1-32 h*$^{-}$	This study
KGY9230-2	*ppc89-mNG:hphMX6 sid4-RFP:kanMX6 ade6-M21X ura4-D18 leu1-32 h*$^{-}$	This study
KGY9231-2	*ppc89(1-707)-mNG:hphMX6 sid4-RFP:kanMX6 ade6-M21X ura4-D18 leu1-32 h*$^{-}$	This study
**Figure 4**		
KGY1283-2	*dma1-mNG:kanMX6 sad1-mCherry:natMX6 ade6-M21X leu1-32 ura4-D18 h*$^{+}$	Lab stock
KGY7740-2	*ppc89-3:kanMX6 dma1-mNG:kanMX6 sad1-mCherry:natMX6 ade6-M21X leu1-32 ura4-D18 h*$^{+}$	This study
KGY8184-2	*ppc89-4:kanMX6 dma1-mNG:kanMX6 sad1-mCherry:natMX6 ade6-M21X leu1-32 ura4-D18 h*$^{+}$	This study
KGY7761-2	*mto1-mNG:kanMX6 sad1-mCherry:natMX6 ade6-M21X ura4-D18 leu1-32 h*$^{+}$	This study
KGY8054-2	*ppc89-3:kanMX6 mto1-mNG:kanMX6 sad1-mCherry:natMX6 ade6-M21X leu1-32 ura4-D18 h*$^{+}$	This study
KGY8124-2	*ppc89-4:kanMX6 mto1-mNG:kanMX6 sad1-mCherry:natMX6 ade6-M21X leu1-32 ura4-D18 h*$^{-}$	This study
KGY9077-2	*ppc89-mCherry:kanMX6 mto1-mNG:kanMX6 ade6-M21X ura4-D18 leu1-32 h*$^{-}$	This study

KGY8719-2	*ppc89(1-707)-mCherry:natMX6 mto1-mNG:kanMX6 ade6-M21X ura4-D18 leu1-32* h^-	This study
**Figure 5**		
KGY7945-2	*ppc89-3-GFP:hphMX6 ade6-M210 ura4-D18 leu1-32* h^-	This study
KGY2603-2	*sid4-GBP-mCherry:kanMX6 ade6-M210 ura4-D18 leu1-32* h^-	Lab stock
KGY7986-2	*ppc89-3-GFP:hphMX6 sid4-GBP-mCherry:kanMX6 ade6-M21X ura4-D18 leu1-32* h^+	This study
KGY7984-2	*ppc89-GFP:hphMX6 sid4-GBP-mCherry:kanMX6 ade6-M21X ura4-D18 leu1-32* h^+	This study
KGY2693-2	*cdr2-GBP-mCherry:kanMX6 ade6-M210 ura-D18 leu1-32* h^-	Lab stock
KGY7987-2	*ppc89-3-GFP:hphMX6 cdr2-GBP-mCherry:kanMX6 ade6-M21X ura4-D18 leu1-32* h^-	This study
KGY15554	*pcp1-GFP:kanMX6 ade6-M21X ura4-D18 leu1-32* h^+	Lab stock
KGY18751	*sid4-mCherry:natMX6 ade6-M21X ura4-D18 leu1-32* h^-	Lab stock
KGY9107-2	*pcp1-GFP:kanMX6 sid4-mCherry:natMX6 ade6-M21X ura4-D18 leu1-32* h^-	This study
KGY9078-2	*pcp1-GFP:kanMX6 sid4-GBP-mCherry:kanMX6 ade6-M21X ura4-D18 leu1-32* h^+	This study
KGY9109-2	*ppc89-4:kanMX6 pcp1-GFP:kanMX6 ade6-M21X ura4-D18 leu1-32* h^-	This study
KGY9099-2	*ppc89-4:kanMX6 sid4-mCherry:natMX6 ade6-M21X ura4-D18 leu1-32* h^+	This study
KGY8600-2	*ppc89-4:kanMX6 sid4-GBP-mCherry:kanMX6 ade6-M21X ura4-D18 leu1-32* h^-	This study
KGY9060-2	*ppc89-4:kanMX6 pcp1-GFP:kanMX6 sid4-mCherry:natMX6 ade6-M21X ura4-D18 leu1-32* h^-	This study
KGY9096-2	*ppc89-4:kanMX6 pcp1-GFP:kanMX6 sid4-GBP-mCherry:kanMX6 ade6-M21X ura4-D18 leu1-32* h^-	This study
**Figure 6**		
KGY9434-2	*ppc89-L756P,I770V-mNG:hphMX6 ade6-M21X ura4-D18 leu1-32* h^-	This study
KGY9573-2	*ppc89-L756P,I770V-mNG:hphMX6 mCherry-cdc15 ade6-M21X ura4-D18 leu1-32* h^+	This study
**Figure S1**		
KGY1288	*spg1-106 ade6-M21X ura4-D18 leu1-32* h^+	Balasubramanian et al., 1998
KGY7114-2	*ppc89-3:kanMX6 spg1-106 ade6-M21X ura4-D18 leu1-32* h^+	This study
KGY7994	*mob1-R4 ade6-M21X ura4-D18 leu1-32* h^-	Yan et al., 2008
KGY8038-2	*ppc89-3:kanMX6 mob1-R4 ade6-M21X ura4-D18 leu1-32* h^+	This study
KGY15086	*plo1-25 ade6-M21X ura4-D18 leu1-32* h^+	Bahler et al., 1998
KGY7062-2	*ppc89-3:kanMX6 plo1-25 ade6-M21X ura4-D18 leu1-32* h^+	This study
KGY1234	*sid4-SA1 ade6-M21X ura4-D18 leu1-32* h^-	Balasubramanian et al., 1998
**Figure S2**		
KGY11662	*cdc11-GFP:kanMX6 sad1-mCherry:kanMX6 ade6-M21X leu1-32 ura4-D18* h^-	Lab Stock

KGY7855-2	*ppc89-3:kanMX6 cdc11-GFP:kanMX6 sad1-mCherry:kanMX6 ade6-M21X ura4-D18 leu1-32 h^+*	This study
KGY8190-2	*ppc89-4:kanMX6 cdc11-GFP:kanMX6 sad1-mCherry:kanMX6 ade6-M21X ura4-D18 leu1-32 h^-*	This study
KGY8187-2	*mto2-mNG:kanMX6 sad1-mCherry:natMX6 ade6-M21X leu1-32 ura4-D18 h+*	This study
KGY8723-2	*ppc89-3:kanMX6 mto2-mNG:kanMX6 sad1-mCherry:natMX6 ade6-M21X leu1-32 ura4-D18 h^+*	This study
KGY8225-2	*ppc89-4:kanMX6 mto2-mNG:kanMX6 sad1-mCherry:natMX6 ade6-M21X leu1-32 ura4-D18 h+*	This study
**Figure S3**		
KGY8029-2	*pcp1-GFP:kanMX6 sad1-mCherry:natMX6 ade6-M21X ura4-D18 leu1-32 h^-*	This study
KGY8175-2	*pcp1-GFP:kanMX6 ppc89-4:kanMX6 sad1-mCherry:natMX6 ade6-M21X ura4-D18 leu1-32 h^+*	This study
KGY8033-2	*sfi1-GFP:kanMX6 sad1-mCherry:kanMX6 ade6-M2X6 leu1-32 ura4-D18 h^+*	This study
KGY8031-2	*ppc89-3: kanMX6 sfi1-GFP:kanMX6 sad1-mCherry:kanMX6 ade6-M21X ura4-D18 leu1-32 h^-*	This study
KGY8125-2	*ppc89-4: kanMX6 sfi1-GFP:kanMX6 sad1-mCherry:kanMX6 ade6-M21X ura4-D18 leu1-32 h+*	This study
KGY9084-2	*ppc89-3:kanMX6 pcp1-GFP:kanMX6 ade6-M21X ura-D18 leu1-32 h^-*	This study
KGY9108-2	*ppc89-3:kanMX6 sid4-mCherry:natMX6 ade6-M21X ura-D18 leu1-32 h^+*	This study
KGY9085-2	*ppc89-3:kanMX6 sid4-GBP-mCherry:kanMX6 ade6-M21X ura-D18 leu1-32 h^-*	This study
KGY9079-2	*ppc89-3:kanMX6 pcp1-GFP:kanMX6 sid4-mCherry:natMX6 ade6-M21X ura-D18 leu1-32 h^+*	This study
KGY9074-2	*ppc89-3:kanMX6 pcp1-GFP:kanMX6 sid4-GBP-mCherry:kanMX6 ade6-M21X ura-D18 leu1-32 h^-*	This study
